# Unveiling antibacterial and antioxidant activities of zinc phosphate-based nanosheets synthesized by *Aspergillus fumigatus* and its application in sustainable decolorization of textile wastewater

**DOI:** 10.1186/s12866-023-03054-x

**Published:** 2023-11-18

**Authors:** Reyad M. El-Sharkawy, Mohamed H. H. Abbas

**Affiliations:** 1 Botany and Microbiology Department, Faculty of Science, Benha University, Benha, 13511 Egypt; 2 Soils and Water Department, Faculty of Science, Benha University, Benha, Egypt

**Keywords:** *Aspergillus fumigatus*, Biosynthesis, Ecofriendly, Nanosheets, Antibacterial, Antioxidant, Water pollution, Adsorption, Decolorization, Textile dye

## Abstract

**Background:**

The development of an environment-friendly nanomaterial with promising antimicrobial and antioxidant properties is highly desirable. The decolorization potentiality of toxic dyes using nanoparticles is a progressively serious worldwide issue.

**Methods:**

The successful biosynthesis of zinc nanoparticles based on phosphates (ZnP-nps) was performed using the extracellular secretions of *Aspergillus fumigatus*. The antibacterial activity of the biosynthetic ZnP-nps was investigated against Gram-negative bacteria and Gram-positive bacteria using the agar diffusion assay method. The antioxidant property for the biosynthetic nanomaterial was evaluated by DPPH and H_2_O_2_ radical scavenging assay.

**Results:**

Remarkable antibacterial and antiradical scavenging activities of ZnP-nps were observed in a dose-dependent manner. The minimum inhibitory concentration (MIC) for *Staphylococcus aureus*, *Pseudomonas aeruginosa*, and *Escherichia coli* was 25 µg/ml, however, the MIC for *Bacillus subtilis* was 12.5 µg/ml. The maximum adsorptive performance of nanomaterial was respectively achieved at initial dye concentration of 200 mg/L and 150 mg/L using methylene blue (MB) and methyl orange (MO), where sorbent dosages were 0.5 g for MB and 0.75 g for MB; pH was 8.0 for MB and 4.0 for MO; temperature was 30 °C; contact time was 120 min. The experimental data was better obeyed with Langmuir’s isotherm and pseudo-second-order kinetic model (R^2^ > 0.999). The maximum adsorption capacity (q_max_) of MB and MO dyes on nanomaterial were 178.25 mg/g and 50.10 mg/g, respectively. The regenerated nanomaterial, respectively, persist > 90% and 60% for MB and MO after 6 successive cycles. The adsorption capacity of the prepared zinc phosphate nanosheets crystal toward MB and MO, in the present study, was comparable/superior with other previously engineered adsorbents.

**Conclusions:**

Based on the above results, the biosynthesized ZnP-nanosheets are promising nanomaterial for their application in sustainable dye decolorization processes and they can be employed in controlling different pathogenic bacteria with a potential application as antiradical scavenging agent. Up to our knowledge, this is probably the first study conducted on the green synthesis of ZnP-nanosheets by filamentous fungus and its significant in sustainable dye decolorization.

**Supplementary Information:**

The online version contains supplementary material available at 10.1186/s12866-023-03054-x.

## Introduction

Nanomaterials have evidenced widespread significance in various fields including environmental protection, chemistry, agriculture, sensors, catalysts, food-packing, textile, electronics and biomedical [1–5]. Top-down and bottom-up techniques are often employed in the nanoparticle’s synthesis, exhibiting several demerits such as polluting, inefficient and time consuming [6–11]. The development of trustworthy, and eco-friendly routes for manufacturing various types of nanomaterials has been growing with a remarkable importance in recent years [11–17]. To circumvent the limitation of traditional approaches used in nanomaterial synthesis, the green synthetic technologies with minor environmental influence are quite a modern approach [9, 10, 12, 14, 15, 18–20]. The green synthetic processes for nanomaterials production, which is mainly based on bioactive agents like plants, bacteria, fungi, and actinomycetes are of promising environmental impact. This technique has several advantages including simplicity, less time-consuming, non-toxicity of the formed by-products, cost-effectiveness, good stability of the produced nanoparticles, environment friendly as the risk of contamination is lower, and easily scaled up for large scale production [5, 14, 17, 21–24]. Amongst the developed nanomaterial, layered nanomaterials have received great attractiveness due to their high flexibility and diversity, wide applicability, exchangeable, interlayer and intercalation reaction potential, their regulating potentials, and binding capacities. The remarkable delamination capabilities of these layered nanomaterials are highly desirable for new, different and vast applications [1, 5].

Zinc-based micro/nanoparticles are deemed as a multifunctional material with vast promising applications. They are fabricated using various preparation methods, and exhibited various morphological features including sheet-like, hexagonal, rods, globular and three-dimensional flower like structures [1–3, 20]. They have attracted the scientific community since they have unique physicochemical characters and a reasonable price. Compared with the developed nanoscale materials, zinc-based nanoparticles are the most broadly employed nanomaterial for drug delivery, dental applications, cancer therapy, wound healing, and antimicrobial agents [14, 15, 20, 25]. Amongst these zinc-based micro/nanostructures, zinc nanoparticles-based phosphates have received significant attraction and was examined for combating multidrug resistance (MDR) in pathogenic bacteria, anticancer, and antioxidant activities [2, 3, 20]. However, the biomedical efficiency of zinc nanoparticles-based phosphates as antibacterial, antioxidant, and cytotoxicity activities are slightly investigated. The high dose of zinc has antimicrobial effects, and hence may act as an alternative source to old-style antibiotics. This may reduce the extent of antibiotic resistance characters among different microbial pathogens [2, 5, 20, 26].

Water pollution resulting from the discharge of toxic dyes into the natural water environment has become a serious issue for developing and underdeveloped economics. Management of water contamination caused by textile dyes is one of the most problematic issues in the new century [4, 5]. Synthetic/toxic dyes can emerge from several industries including leather, textile, cosmetic, printing, plastics, and pharmaceuticals. It is estimated that 8–20% of the total dyestuffs are released into the water environment through their synthesis and textile industry [27, 28]. The discharge of these toxic dyes into water bodies is unacceptable and may lead to serious harmful effects on environment, health, and aquatic life [4, 5, 29]. Unquestionably, the decolorization and detoxification of these synthetic dyes prior to their discharge is of great interest.

Regarding their complex structure and non-degradable nature, the clean-up technique of toxic dyes is difficult and requires collaboration of several technologies. The decolorization of textile dye effluents has achieved by several physical and chemical methods, i.e., ion-exchange, flocculation, coagulation, and precipitation [4, 5, 29–31]. The above ways are still ineffective, expensive and re-contaminated the environment by the produced toxic substances. Hence, the development of ecofriendly and effective methods is quiet an open challenge for removing toxic dyes from water [4, 5, 32, 33].

Biological treatment of wastewater, particularly biosorption, is comparatively better for dyes removal from aqueous solutions than other techniques [4, 5, 34, 35]. Most studies on toxic dyes treatments are applying various types of adsorbents including rice husk [36], fava bean peel [37], banana pith [38], zeolites, ZnO/Chitosan [28] and particularly nanomaterial synthesized using microbial byproduct [35, 39]. Several techniques are applied for synthesis of nanomaterial as promising adsorbents for removing water contaminant including sol–gel [40] and co-precipitation [23]. However, these methods have many drawbacks, i.e., high temperature, and pressure as well as generation of hazardous substances.

Currently, the biological route of novel biosorbent synthesis has a great potential to eradicate toxic materials from industrial effluents and water systems [5]. High adsorption capacity, slow kinetics, easy desorption, and high reusability are required charterers for the sufficient adsorption of pollutants as reported by [4, 5, 39]. Most of the biosynthetic nanomaterial were utilized in the adsorptive elimination of toxic heavy metals [33]. However, only a few reports have studied the potentiality of adsorptive removal of toxic dyes using the biosynthesized nanosheets powder.

The novelty of the undercurrent investigation is articulated as a remarkable improvement in sustainable decolorization of different textile dyes from aqueous solutions in a superior removal capacity with promising antibacterial and antioxidant activities. The purpose of this study was achieved by the following objectiives: (i) biosynthesize the zinc phosphate nanosheet crystal [Zn_3_(PO_4_)_2_] with lower environmental influence via green synthesis approach by harnessing the exo-metabolites of *Aspergillus fumigatus* for the first time, (ii) evaluate nanoproduct for its antibacterial potentiality against Gram-negative ad Gram-positive pathogens and their antioxidant activities toward DPPH and H_2_O_2_ free radicals and (iii) investigate a sustainable eradication technique for removing textile dyes from polluted water using the prepared nanomaterial in a high removal rate and a superior adsorption capacity without elimination of a secondary contamination.

## Materials and methods

**Fig. 1:**
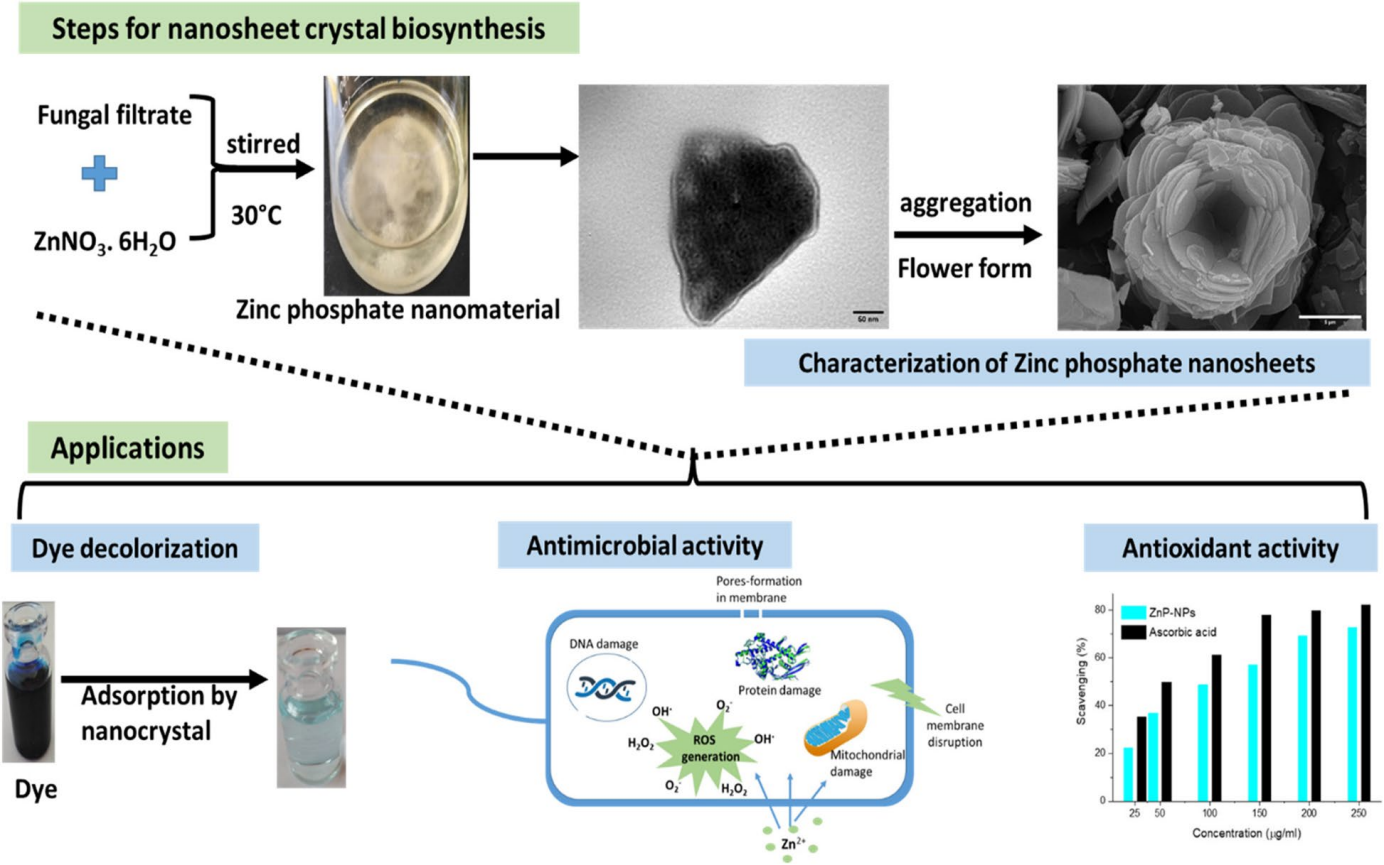
Schematic diagram for the biosynthesis of zinc phosphate-based nanosheets using the exometabolites of *A. fumigatus* with their potential antibacterial, antioxidant and dye decolorization nanomaterial

A schematic diagram presenting the overall process for the biosynthesis of zinc phosphate-based nanosheets using the exometabolites of *A. fumigatus* with their evaluation as promising antibacterial and, antioxidant nanomaterial and as a potential dye decolorization adsorbent as illustrated in Fig. 1.

### Chemicals

All chemicals in the current study were purchased from Sigma-Aldrich (Cairo, Egypt) and are of analytical grade. Deionized water was employed for preparing various solutions.

### Microorganism

The fungal isolates in the current study were obtained from various soil samples collected from Benha, Egypt. The fungal isolation process was performed according to [5, 19]. Among the tested fungi, the fungal isolate EG-RE-ZnP-nps, was selected for the synthesis of zinc phosphate-based nanoparticles. The fungus was grown on a medium containing % (Glucose 1.0; yeast extracts 0.06; (NH_4_)_2_SO_4_; 0.1; K_2_HPO_4_ 0.2; 0.1; MgSO_4_.7H_2_O 0.01; KH_2_PO_4_ 0.07; pH 6.5) at 28 ± 1 °C for 5 days with shaking at 150 rpm. Fungal beads were removed by filtration, subsequently, the developed filtrate was then centrifuged for 10 min at 5,000 × g at 4 °C. The clarified supernatant was kept at -20 °C for further work.

### Molecular identification of fungal isolate mediated ZnP-nps synthesis

The selected fungal isolate was grown on Czapek’s-Dox agar medium at 28 ± 1 °C for 5 days, and the obtained colony was daily examined and identified according to [41, 42] to species level. The morphological and microscopical identity of the EG-ZnP-nps isolate was confirmed based on the sequence of 18S-ITS1-5.8S-ITS2-28S region [4, 42]. Briefly, fungal genomic DNA, which was extracted from *A. fumigatus*, was used as template for PCR amplification with ITS-1 primer (5′– TCC GTA GGT GAA CCT GCG G –3') and ITS–4 primer (5'–TCC TCC GCT TAT TGA TAT GC–3') which was separated on 1% agarose gel [42]. For sequencing alignment, the obtained nucleotide sequence was submitted to the GenBank Basic Local Alignment Search (BLASTn) Tool (http://www.ncbi.nlm.nih.gov/Blast). While for, phylogenetic tree and molecular evolutionary analyses, the obtained ITS sequence with the GenBank closely related sequences were imported into MEGA-X V 11.0. and the phylogenetic tree was then conducted by using the Neighbor-Joining method at confidence level of each branch of 1000 bootstrap replication [42, 43].

### Synthesis of ZnP-nanosheets

An appropriate concentration of ZnNO_3_.6H_2_O was mixed at a ratio of 1:1 with the fungal supernatant for the green synthesis of ZnP-nanosheet crystal and the mixture was stirred for 2 h. A white gel was produced after 5 h, which was aged for 2 days at 30 ± 1 °C. The resultant nanoparticles were recovered via centrifugation for 10 min at 5,000 × g. The nanosheets crystal was pooled, washed with distilled water, dried, and stored for further utilization in adsorptive experiments [2, 20].

### Characterization of ZnP-nanosheets

X-ray powder diffraction (XRD) pattern was conducted via MiniFelx 300/600 X-ray, USA using CuKα (λ = 1.541 Å) radiation operated at 40 kV and 15 mA and the scan rate of 2ϴ = 0°-85°. The morphology of the biosynthetic nanosheets crystal was examined with SEM (JEOL JSM-6510LV, Tokyo, Japan) with 15 kV acceleration voltage. The as-prepared samples were sprayed with gold under vacuum and low pressure, dried and then observed by SEM. The elemental composition of nanomaterial was determined via energy dispersive X-ray spectrometer (EDX) mounted on SEM. TEM analysis was observed using a Philips EM-420 microscope with 200 kV acceleration voltage. Samples were positioned on a carbon-coated grid, vacuum-dried and then used for TEM snapshots. In Fourier transform infrared (FTIR) spectroscopy, the functional groups existing in the dried sample were recorded at resolution of 4.00 cm^−1^ under the spectral range from 500 to 4000 cm^−1^ [2, 3, 20, 44].

### In vitro evaluation of antibacterial activity

The antibacterial activity of the biosynthetic ZnP-based nanostructure was investigated against Gram-negative bacteria (*Escherichia coli* and *Pseudomonas aeruginosa*) and Gram-positive bacteria (*Bacillus subtilis* and *Staphylococcus aureus*) by the agar diffusion assay method [20, 45] with some modification. In brief, for each bacterial suspension 50 µl of overnight bacterial broth were separately seeded into Muller-Hinton agar (MHA, 100 ml), shake well, and subsequently poured in sterilized dishes underneath aseptic conditions. Different concentrations of ZnP-based nanomaterial solution (100 µl, 100–6.25 µg/ml) were introduced into 0.7 cm-well in the inoculated MHA plates. These loaded plates were incubated for overnight at 37℃ and the diameters of the inhibitory zones were measured in mm. The inhibitory zones were compared to streptomycin as a reference antibiotic under the same conditions.

### Structural changes in the morphology of bacterial cell *B. subtilis*

The morphological changes of *B. subtilis* treated with minimum bactericidal (MBC) dose of ZnP-nps synthesized by green approach was observed using scanning electron microscope (SEM, JEOL JSM-6510LV microscope, Japan). The bacterial culture subject to 24 h of ZnP-nps treatment were collected via centrifugation for 10 min at 8,000 rpm. The collected pellets were rinsed by sterile distilled H_2_O. The bacterial preparation was prepared for SEM visualization according to [46] with slight modifications. The structural changes of the treated cells were compared with the untreated cells (control) which are performed using the same conditions.

### Evaluation of antioxidant activity

#### DPPH assay

The antioxidant property for the biosynthetic ZnP-based nanomaterial and standard ascorbic acid was monitored by using DPPH radical scavenging assay [22, 47]. Different concentrations of the investigated sample and ascorbic acid (25–250 µg/ml) were mixed in equal volume with freshly prepared ethanolic DPPH (0.1 mM). The preparations were thoroughly vortex, incubated at ambient temperature in dark for 30 min under shaking conditions, and then the absorbance was monitored at 517 nm. In parallel, ethanolic DPPH without ZnP-nps and ethanol alone were respectively used as control and blank preparation which were performed under the same conditions. DPPH scavenging inhibition was calculated from the following formula:

$$\mathrm{Inhibition }\left(\mathrm{\%}\right)= \frac{{B}_{C}- {B}_{S}}{ {B}_{C}} \times 100$$

where, ${B}_{C}$ designate the absorbance of control and ${B}_{S}$ designate the absorbance of sample.

### H_2_O_2_ radical scavenging assay

For determination of H_2_O_2_ radical scavenging activity for the biosynthetic ZnP-based nanomaterial and ascorbic acid as reference standard, the scavenging activity was assayed according to [48, 49] with slight modification. In brief, 0.3 ml phosphate buffer (pH 7.4, 50 mM), 0.6 ml of 2 mM H_2_O_2_ solution in phosphate buffer (50 mM, pH 7.4) and different concentrations of the tested nanopowder (25–250 µg/ml) in the same buffer were thoroughly mixed for 15 min, and subsequently the absorbance was monitored at 230 nm. Blank containing phosphate buffer without H_2_O_2_ was used. The concentration of H_2_O_2_ scavenging was detectable by the equation mentioned for DPPH assay.

### Catalytic decolorization of MB and MO using ZnP-nansheet crystals

#### Adsorption experiments

The influence of adsorbent dosage (0.25–1.25 g), pH (2.0–10.0), initial dye concentration (10–300 mg/L) and contact time (5–240 min) on the cationic and anionic dyes removal efficiency using ZnP-based nanosheets prepared by the fungal filtrate of *A. fumigatus* was investigated according to [28, 39, 50] with some modification. All experiments were conducted at 150 rpm. At the end of the experiments, the final concentration of MB and MO dyes in the preparations were estimated using a standard curve of each of the individual dye. The removal efficiency (R%) and adsorption capacity (q_e_) at equilibrium were respectively determined by Eqs. (1) and (2).

1 $$\mathrm{R }(\mathrm{\%}) = \frac{({C}_{0}- {C}_{f})}{{C}_{f}} \times 100$$

2 $$\mathrm{qe }= \frac{{(C}_{0}- {C}_{e}) V}{M}$$

wherein *C*_*0*_, *C*_*f*_ and *C*_*e*_ represent the initial, final and equilibrium dye concentration, respectively; *M* and *V* are sorbent concentration (g) and the adsorbate volume (L), respectively.

The adsorption (Langmuir and Freundlich) isotherms are harnessed to describe the experimental data obtained and were determined using the, Eqs. (3) and (4), respectively:

3 $$\mathrm{q }= \frac{{q}_{max} b {C}_{f}}{1+b {C}_{f}}$$

4 $$q\;=\;KF\;C1/n$$

where q_max_ represents the maximum biosorption (mg/g); b is the sorption equilibrium constant; K_*F*_ (mg/g) is the sorption equilibrium capacity and n is the sorption intensity.

The evaluation of adsorption kinetics was performed by employing the pseudo-first and second- order kinetics as described by the Eqs. (5) and (6).

5 $$\mathrm{ln}\left({q}_{e}- {q}_{t}\right)= \mathrm{ln}{q}_{e}- {K}_{1}t$$

6 $$\frac{t}{{q}_{\mathrm{e}}}= \frac{1}{{K}_{2} {q}_{\mathrm{e}}^{2}}+\frac{1}{{q}_{\mathrm{e}}}$$

where ${K}_{1}$ and ${K}_{2}$ denote the rate constant of pseudo-first-order biosorption and pseudo-second-order biosorption at equilibrium.

### Desorption experiments

In order to assess long term applications of the prepared ZnP-based nanosheets, the regeneration of adsorbent was investigated in eight consecutive cycles. A desirable amount of the sorbent (as 0.5 g for MB and 0.75 g for MO) was inserted in 100 ml of the dye solution (as 100 mg/l) at 30 °C, pH: 8.0 (MB) and pH 4.0 (MO), 150 rpm, and 120 min). The nanosheets were harvested via filtration, washed with deionized water, and the concentration of the residual dye in preparations was determined. At the end of each adsorption cycle, the nanomaterial-loaded dye was treated with 50 ml of 0.1 M HCl (stripping solution) at 30 °C, 200 rpm, and 120 min). The desorbed nanomaterial was collected, and the dye concentration was measured as described earlier. The nanosheet crystals were washed at frequent times using distilled water to neutrality, dried and subsequently re-employed in the biosorption-desorption process. The desorption efficiency was measured by Eq. (7):

7 $$\mathrm{Desorption \,efficiency }(\mathrm{\%}) = \frac{{C}_{d}}{{C}_{s}} \times 100$$

where C_d_ and C_s_ (mg g^−1^) denote the amount of dye desorbed and initially adsorbed on the biosorbent.

### Statistical analysis

All the experiments were accomplished in triplicate and their mean values were displayed with the standard deviation (S.D.).

## Results and discussion

### Molecular characterization of the fungal isolate mediated synthesis of zinc nanoparticles-based phosphates

**Fig. 2:**
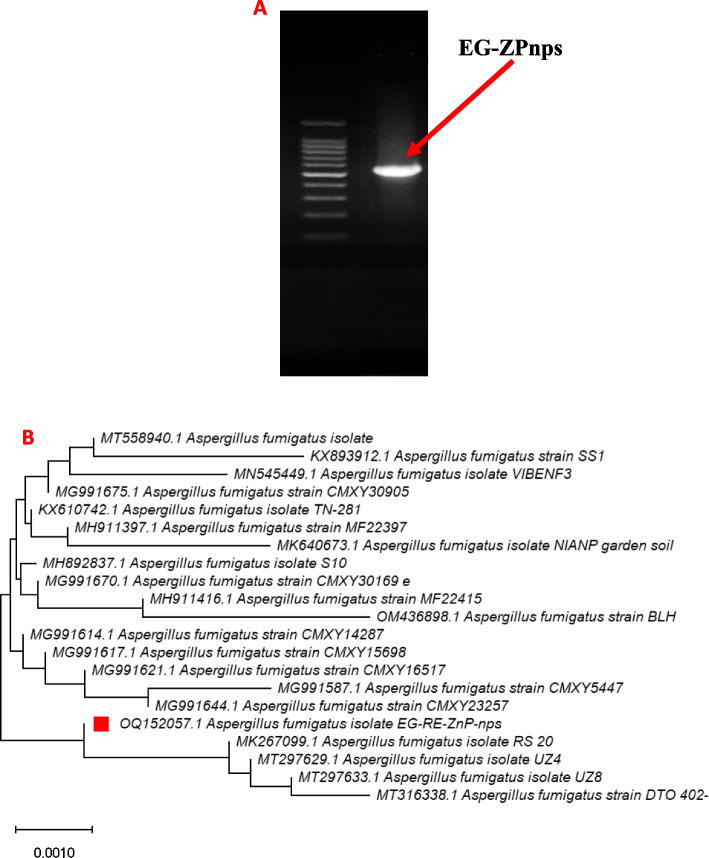
**A** PCR amplicon of 18S-ITS1-5.8S-ITS2-28S region of the fungal strain EG-RE-ZnP-nps. **B** Neighbor-Joining tree of the *A. fumigates* EG-ZnP-nps sequence retrieved from the current work with the NCBI sequences using MEGA-X 11 software. The symbol (  ) represents the ITS sequence of the fungal strain in this work. For each nucleotide site, the bar length designates 0.0010 substitutions

The biosynthetic zinc phosphate nanosheet crystals were successfully performed by harnessing the biomolecules produced in the fungal filtrate of the EG-RE-ZnP-nps isolate. To verify the morphological identification, the fungal isolate mediated ZnP-nanoparticles synthesis was genetically identified based on its rRNA-ITS fragment region. The PCR amplicon was approximately 556 bp (Fig. 2A, uncropped gel in Figure S1). Applying BLAST search, reveal that the amplicon was closely related to *Aspergillus fumigatus* and deposited on GenBank database with accession number OQ152057.1. The isolate *A. fumigatus* EG-RE-ZnP-nps displaying a similarity percentage of 99% with *A. fumigatus* isolates with accession numbers MK267099.1, MT297629.1 and MT316338.1 with 100% query coverage and zero E-value. The phylogenetic tree of *A. fumigatus* and closely related GenBank sequences was constructed using the MEGA-X neighbor-joining method (Fig. 2B).

### Characterization

**Fig. 3:**
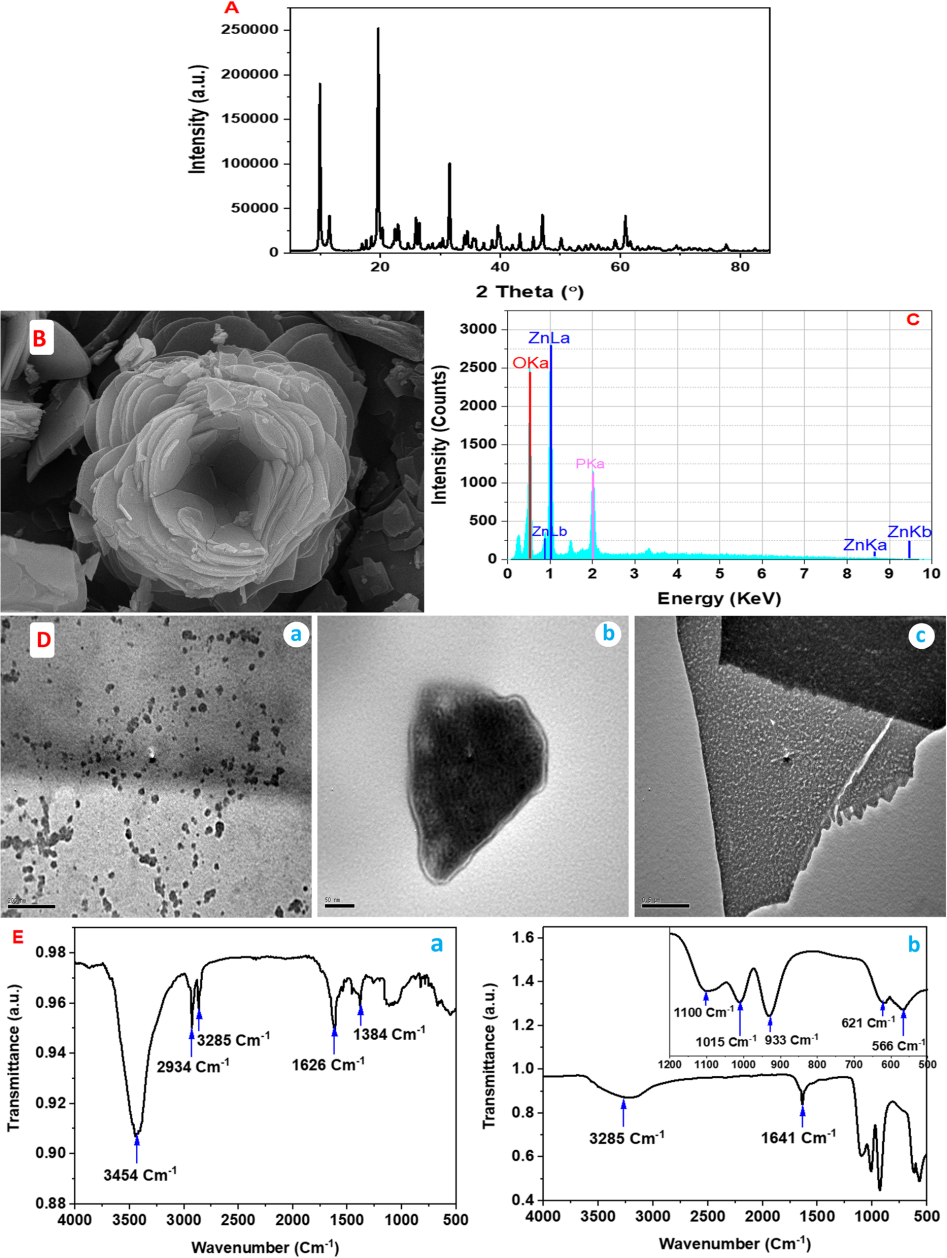
**A** Diffraction pattern, **B** SEM image, **C** EDX spectrum of the zinc phosphate-based nanosheet synthesized by *A. fumigatus*, **D** TEM images of the prepared sheets sampled through the initial stage (a) and the growth of sheet crystals (b,c), **E** FTIR of the fungal filtrate (a) and the zinc phosphate nanopowder (b) synthesized by using the exo-metabolites of *A. fumigatus*

The XRD analysis of the as-prepared ZnP-nanostructure is shown in Fig. 3A. The crystalline structure of the representative sample using the exo-metabolites of *A. fumigatus* allowed the identification of sample by comparing with Powder Diffraction Files (PDF). The diffraction reflections were consistent with the standard files of Zn_3_(PO_4_)_2_ × 4 H_2_O (PDF no. 9012092). Peaks of impurities were not observed, denoting the purely crystalline powder. Therefore, the spectra confirmed the successfully biosynthesize of zinc nanoparticles based on phosphates. The successful biosynthesis of ZnP-nanoparticles has been reported by [1, 44, 51].

SEM imaging of the resultant ZnP-nanosheets after 2 days is shown in Fig. 3B. The samples present a flower-like zinc phosphate crystal structure with an irregular nanosheets morphology, multi-faceted crystals with a high degree of agglomeration Fig. 3B. Agglomeration may be due to a narrow-space between nanoparticles and a high surface energy of zinc phosphate nanomaterial. [44] confirmed the occurrence of a transitional morphology in zinc phosphate crystals from flower-like structure, prism, sheets, and multi-faceted self-assembled crystals to microfibers during a minor alteration in the pH of the reaction system. Similar results were made by [44, 51–53]; during the investigations of zinc phosphate nanoparticles.

The biosynthetic nanomaterial was characterized by EDX analysis (Fig. 3C). EDX of the nanomaterial revealed the presence of strong peaks of Zn, O and P. No other elements were found. Therefore, EDX micrograph confirmed the purity of the ZnP-nanocrystal which was synthesized by the exometabolites of *A. fumigatus*.

TEM snapshots of the *A. fumigatus* synthesized zinc phosphate-based nanosheets are shown in Fig. 3D. The magnification of Zn_3_(PO_4_)_2_ × 4 H_2_O sheet crystals during the initial nucleation (Fig. 3D-a) and consequent growth of ZnP-nanocrystals using filtrate of *A. fumigatus* is illustrated in Fig. 3D-b,c*.* During the formation mechanism of flower-like zinc phosphate crystal structure, large number of nearly spherical nanoparticles are generated which revealed the formation of primary noncrystalline nuclei nanoparticles and showed evenly an extent of agglomeration. The zinc phosphate nuclei continued to grow and hence generated uniform crystals (first stage). At the second stage, small sheets were formed. These sheets are spontaneously self-assembled and eventually constructed into a flower-like zinc phosphate crystal (third stage). Our findings agree with [20, 44, 53] who evinced that zinc phosphate nanoparticles are produced using cationic ammonium salts and both nucleation and growth of ZnP-nanoparticle crystals was obtained during the progress of nanoparticles synthesis. At initial stage, the average size of the biosynthetic zinc phosphate nanoparticles using *Enterobacter aerogenes* was found to be 30–35 nm [51].

FTIR spectrum of the fungal filtrate and the biosynthetic ZnP-nps are presented in Fig. 3E-a,b. The broad bands at 3485 cm^−1^ and 3285 cm^−1^ have been identified as a band of the O–H stretching vibration. Considerable bands produced at 2919 cm^−1^ and 2853 cm^−1^ were for C–H or O–H stretching vibration, respectively. Bands produced at 1633 cm^−1^ (C = C or C = O stretching vibration), 1641 cm^−1^ (H–O–H stretching vibration of H_2_O molecules), 1375 cm^−1^ and 1255 cm^−1^ (C–O bending vibration) were identified. The corresponding ones produced at 1100 cm^−1^, 1015 cm^−1^, 933 cm^−1^, 621 cm^−1^, and 566 cm^−1^ designate the synthesis of zinc phosphate nanosheet (Fig. 3E-b inset). The FTIR results revealed the zinc phosphate nanosheet crystals contains crystal water. In addition, the spectral studies propose that the functional groups (carbonyl and hydroxyl groups) have considerable affinity to bind with metal during the aged ZnP-nanosheet crystal [54]. Therefore, the FTIR spectra affirmed the capping/coating and partaking of biomolecules from fungal filtrate on the surface of the zinc phosphate nanocrystal via different functional groups.

The formation of ZnP-nanosheets crystal may be occurred by ionic-dipolar interaction through the bind of zinc ions with negatively charged groups (hydroxyl and carbonyl groups) in the fungal filtrate, forming a nucleating point for the growing zinc phosphate crystal where phosphate radicals were then introduced into the mixture. The creation of zinc phosphate nanoparticles complex may also be emerged by firstly forming ionic bond between phosphate radicals and –OH groups (via esterification) in the fungal filtrate and Zn^2+^ ions were then trapped on PO_4_^3−^. The produced zinc phosphate nuclei were subsequently permitting multistage crystallization of Zn_3_(PO_4_)_2_ × 4 H_2_O crystals. It is believed that the agglomeration of ZnP-nps may arise from steric hindrance and electrostatic effect of the prepared nuclei. While condensed particles were obtained by centrifugation [53, 55]. The formation of ZnP-nanosheets is controlled in the pH range of 3.0–7.0, whereby the H^+^ concentration is not fewer than OH^−^ concentration [56]. In the hydrolytic reaction where fungal filtrate present with various functional groups, the concentration of PO_4_^3−^ anions will be decreased and the reaction will move to the right-hand side of the reaction. Subsequently, the phosphate anions will choose to react with Zn^2+^ ions, forming the zinc phosphate as illustrated by the following Eq. 8:

8 $$\mathrm{PO4}^{3-}+\mathrm{NH}4++\mathrm{Zn}^{2+}+\mathrm H2\xrightarrow[\text{exometabolites}]{\text{Fungal}}\mathrm{Zn}3(\mathrm{PO}4).4\mathrm H2\mathrm O(\mathrm s)$$

### Antibacterial assessment of the biosynthetic ZnP-nanomaterial

The rapid emergence of microbial resistance toward conventional antibiotics due to the overuse and misuse of different antibiotics has imposed a critical threat to human life, hindering the rapid diagnosis and treatment of patients. Hence, uncovering new nanoparticles for combating multidrug resistance property in pathogenic bacteria is extremely required. The antibacterial efficiency of the biogenically synthesized ZnP-based nanomaterial was evaluated using agar diffusion assay. The bacterial growth inhibition was assayed based on determining the diameter of inhibition zone, relating to the corresponding concentration of ZnP-nps.

**Fig. 4:**
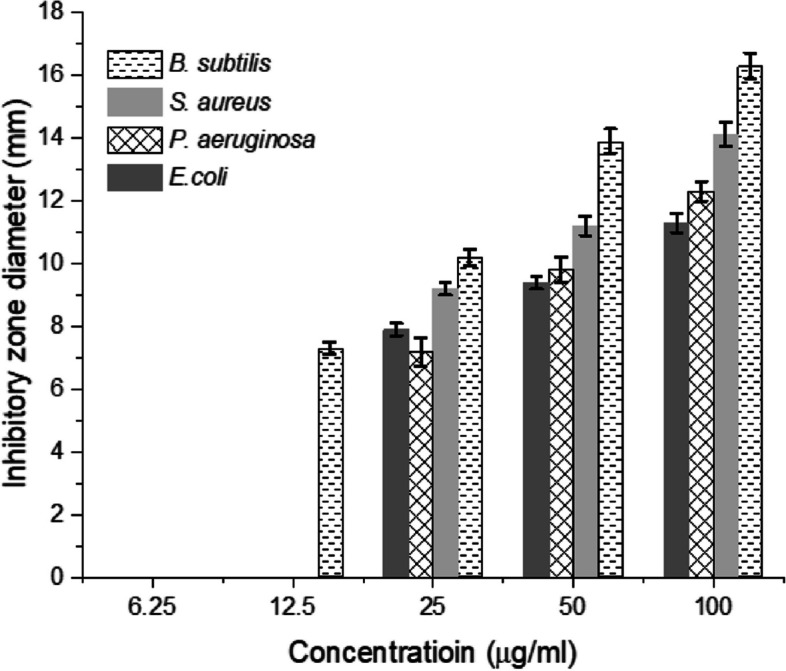
Antibacterial activity of the ZnP-based nanosheets against the Gram-positive and Gram-negative bacteria (a). Results are presented as means ± standard deviations

The antimicrobial effect of various nanomaterials synthesized by green approach is mainly dependent on their physicochemical properties [20, 57]. The antibacterial activity of ZnP-nps synthesized by *A. fumigatus* strain EG-RE-ZPnps against the investigated Gram-positive and Gram–negative bacterial pathogens was found to be in a concentration-dependent manner, as demonstrated in Fig. 4. The examined bacterial pathogens showed plausible fluctuations in the diameters of the inhibitory zones, of which *Bacillus subtilis* was the most susceptible strain. At 100 µg/ml of the biosynthetic ZnP-nanosheets, the zone of inhibition was found to be 14.1, 12.3, and 11.3 mm for *S. aureus*, *Pseudomonas aeruginosa*, and *Escherichia coli*, respectively. The minimum inhibitory concentration (MIC) of ZnP-nps for *S. aureus*, *E. coli*, and *P. aeruginosa* was 25 µg/ml, however, the MIC for *B. subtilis* was 12.5 µg/ml. Interestingly, *S. aureus* displayed the lower MIC value, compared to other investigated bacterial strains. Similar findings on the antibacterial activity of ZnP-nanomaterial have been reported by [58, 59].

### Structural changes in the morphology of *B. subtilis* cell

**Fig. 5:**
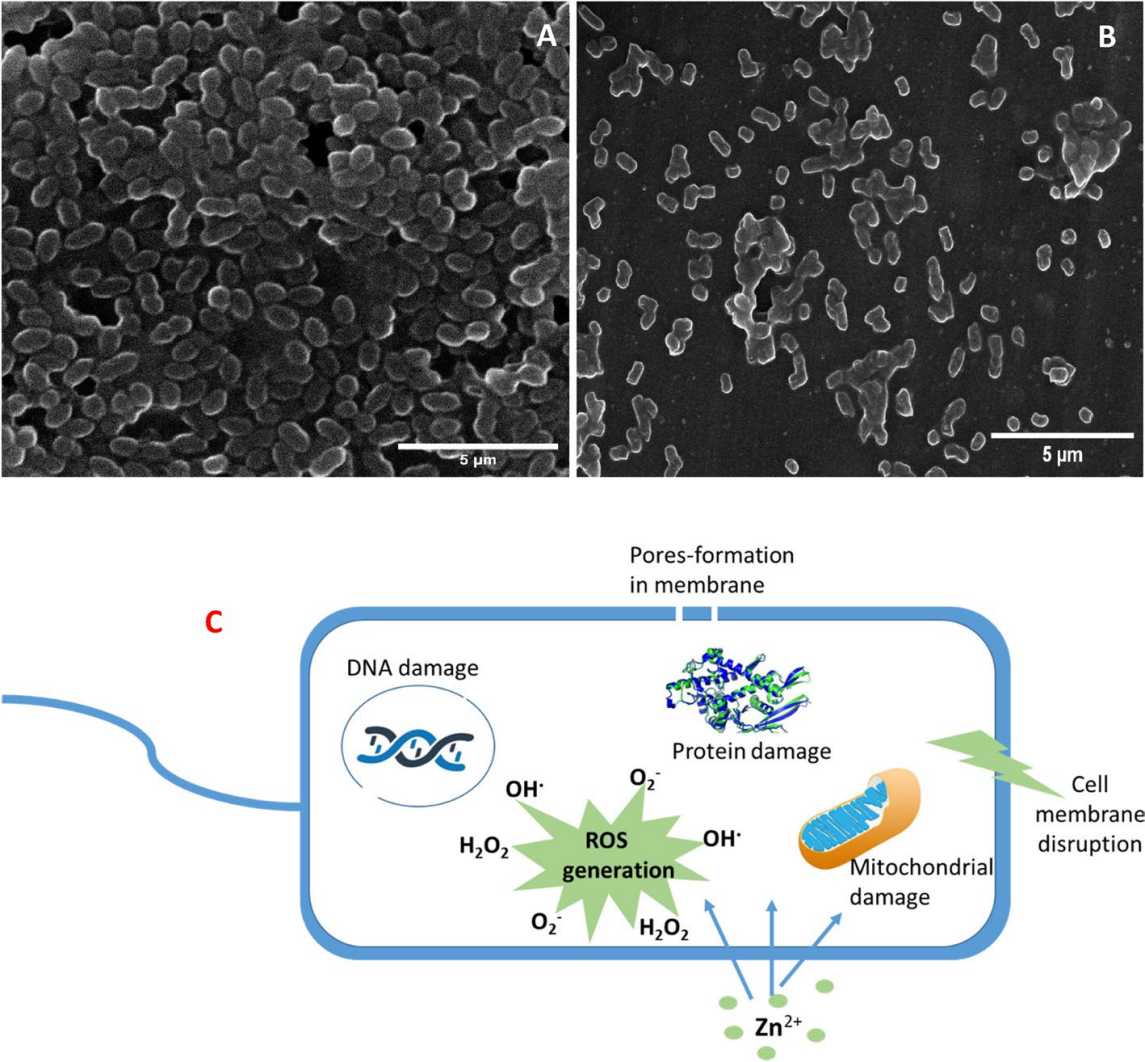
**A** SEM image of overnight *B. subtilis* cells untreated (**A**) and treated (**B**) after incubation 24 h of treatment with ZnP-based nanomaterial synthesized by *A. fumigatus*. SEM image of control cells displaying smooth and undamaged structures. SEM image of treated cells showing disruption and breakdown of the cell and the cell lysis. **C** Schematic mechanism of the proposed antibacterial mode of action of the biosynthetic zinc phosphate-based nanosheets crystal

In order to investigate the manner of action of ZnP-nps fabricated by *A. fumigatus* on the ultrastructure of bacterial pathogens, *B. subtilis* has been chosen as the most susceptible strain, as demarcated from MIC assay. The bacterial culture was treated at MBC dose of ZnP-nps. After incubation at 37 °C for 24 h, the collected bacterial pellets were examined using SEM and compared with bacterial broth culture without nanomaterial as negative control (Fig. 5A). After contact with the nanomaterial, the bacterial cells displayed significant alterations in cell morphology and harm of cells symmetry. A significant wrinkled, degraded, dramatic deformation and finally cell death have been observed in the treated cells from the SEM images (Fig. 5B), in contrary to the control ones which usually show a uniform, regular and contact structure. It has been reported that the morphology of the bacterial cells displayed different structural changes when treated by different nanoparticles as visualized by different imaging techniques including scanning electron microscope (SEM) [20, 57, 59, 60].

The results showed that Gram-positive bacteria were easily inhibited when compared with Gram-negative ones. This may be based on the nature of interactions between the bacterial cell wall and the biosynthetic zinc-based nanomaterial. In addition, the presence of poorly penetrable peptidoglycan layer, and the lipopolysaccharides, as well as the removal ability for foreign materials via a well develop efflux system may also cause a less susceptibility of Gram-negative bacteria toward the zinc-based nanomaterial [20, 26, 59, 61]. The proposed schematic mechanisms of the biosynthetic ZnP-nps were diagrammatically illustrated in Fig. 5C.

Although antibacterial efficiency alone or in combination with conventional antibiotics of various nanoparticles have been affirmed versus different microorganisms, still the precise mechanistic foundation of their antimicrobial action is slight recognized, yet, several researches have been performed to understand their manner of action [59, 60, 62]. Practically, the antimicrobial potential of a specific nanoparticle is mainly dependent on their physico-chemical properties, namely colloidal state, shape, size, surface charge, and concentration. The inhibitory effect of the biosynthetic ZnP-based nanomaterials can be realized on multifaceted mechanisms: (1) The contact interaction of bacterial cell with the zinc-based phosphates nanosheets leads to increase in the negative charge of bacterial cell surface owing to the presence of phosphate ions, (2) An electrostatic communication begins between the positively charged nano-Zn and the bacterial cell wall, (3) adhere, penetrate of Zn^+^ ions inside the microbial cell, and the disruption of cell wall, (4) aggregation on the external side of plasma membrane, (5) discharge of ions into the cell, (6) the production of free radicals and reactive oxygen species, (7) the discharge of Zn^+^ is able to block different vital bacterial enzymes, and can damage bacteria’s DNA, and mitochondria, resulting the leakage of polysaccharide and protein from the cell. Hence, the ZnP- nanosheets crystal exposure to bacterial cell induces the bacteriostatic and bactericidal response in the investigated microbial pathogens [20, 57–60].

### Antioxidant assessment

**Fig. 6:**
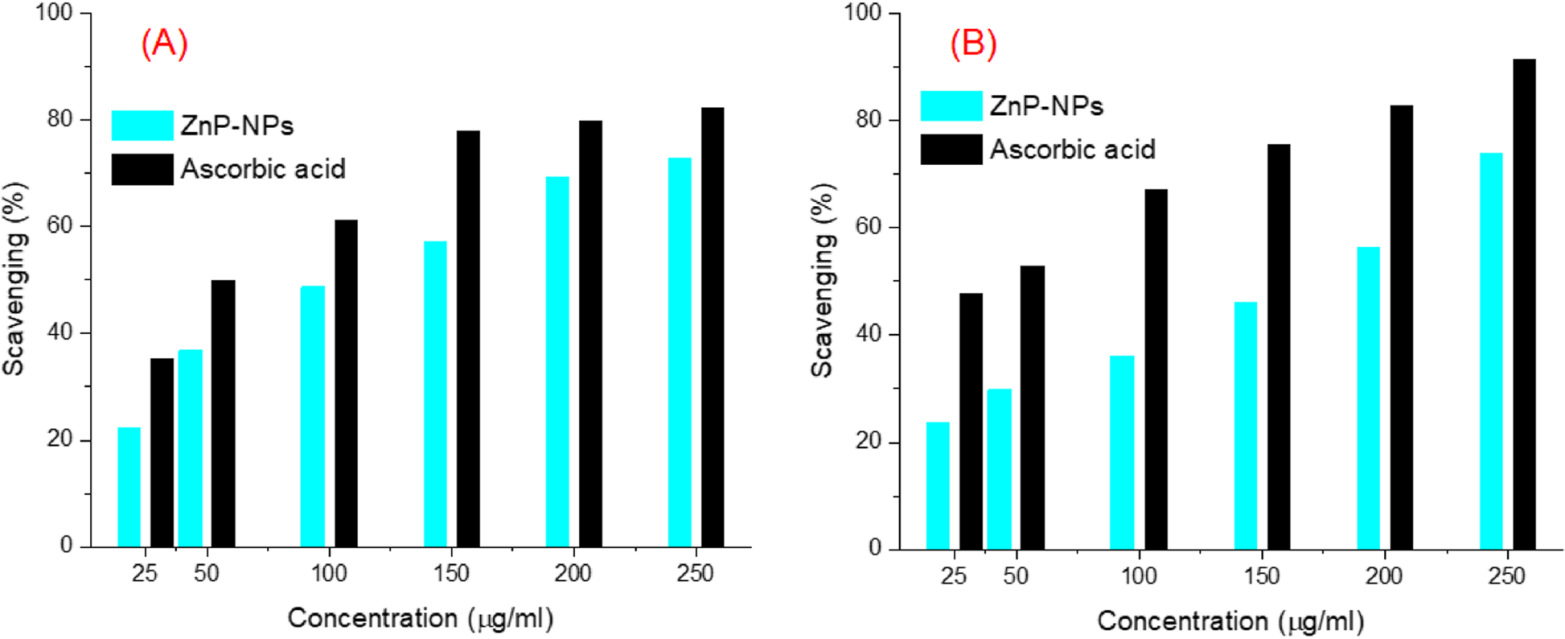
DPPH (**A**), and hydrogen peroxide (**B**) radicals scavenging activity of the ZnP-based nanomaterial prepared by harnessing the biomolecules of *A. fumigatus* using ascorbic acid as respective standard

Antioxidants can protect cells by neutralizing the dangerous free radicals [63]. Herein, the antioxidant activities of the biosynthesized ZnP-based nanomaterial were investigated, and the results were illustrated by Fig. 6**.** The radical scavenging activity was determined by DPPH, and hydrogen peroxide assays. Results of DPPH confirmed that both the biosynthetic ZnP-nanomaterial and ascorbic acid showed antioxidant activity (Fig. 6A**)**. The outcome of the anti-DPPH radical activity of the biosynthetic ZnP-nanomaterial displayed a concentration-dependent manner. On contrary, the antiradical activity of ascorbic acid as a reference standard exhibited significantly higher activity, compared to the tested sample.

The hydrogen peroxide free radical scavenging activity of the *A. fumigatus* synthesized ZnP-based nanostructure was investigated, and the obtained result was illustrated in Fig. 6B. The antiradical scavenging ability of ZnP-based nanomaterial toward H_2_O_2_ radical was remarkably improved with increasing the concentrations of the tested sample. The highest inhibition ability of H_2_O_2_ radical by ZnP-nanomaterial was almost 73.8% at 250 µg/ml of ZnP-nanomaterial which was relatively lower than that of ascorbic acid (91.3%). Similar results have been previously expressed for the antiradical ability of ZnP-nanoparticles [20, 21, 64, 65].

### Catalytic decolorization of dyes from aqueous solutions

To investigate the adsorption characters of ZnP-nps, batch adsorption experiments were carried out by using aqueous solution of methylene blue (MB) and methyl orange (MO) dyes. Methylene blue (MB) and methyl orang (MO) were selected because of their wide applications in various industries. These toxic dyes were introduced as pollutant model for measuring adsorption capacity of the developed nanopowder. A stock solution of both dyes was separately prepared and diluted to the desired concentration.

### Effects of adsorbent dosage

**Fig. 7:**
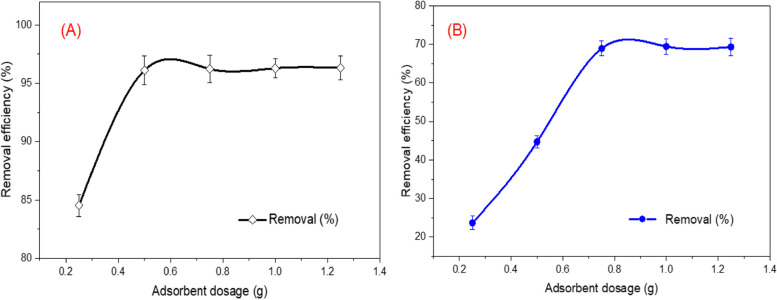
Effect of adsorbent dosage on the removal (%) of MB and MO by zinc phosphate nanosheets synthesized by harnessing the biomolecules of *A. fumigatus* (initial dye concentration: 100 mg/L; solution volume: 100 ml; pH: 8.0 (MB) and pH 4.0 (MO); temperature: 30 °C, agitation speed: 150 rpm; contact time = 120 min)

The influence of initial adsorbent dosage on the sorption of MB and MO was investigated by mixing different amounts (0.25–1.25 g) of the ZnP-nps synthesized by *A. fumigatus* with 100 ml dye solution 100 mg/l. The findings indicated that the removal efficiency of MB and MO by the biosynthesized nanomaterial was gradually increased from 84.53% (0.25 g) to 96.12% (0.5 g) and from 23.67% (0.25 g) to 68.95% (0.75 g), respectively by increasing the sorbent dosage and later remained almost stable. This may be ascribed to the higher active sites available on the adsorbent for dyes molecules as the adsorbent dosage increased and consequently overlapping of adsorption sites. This overlapping reduces the effective specific surface area available for sorbate molecules [39]. In addition, the performance of high adsorbent dosage delivers extra active adsorption sites which become unsaturated after adsorption. Hence, 0.5 g and 0.75 g for MB and MO was selected as optimum for further experiments (Fig. 7 A,B), respectively. Similar findings have been tracked in the previous literatures, connected to the removal of different dyes using various nanomaterials [39, 66, 67].

### Effects of pHs

**Fig. 8:**
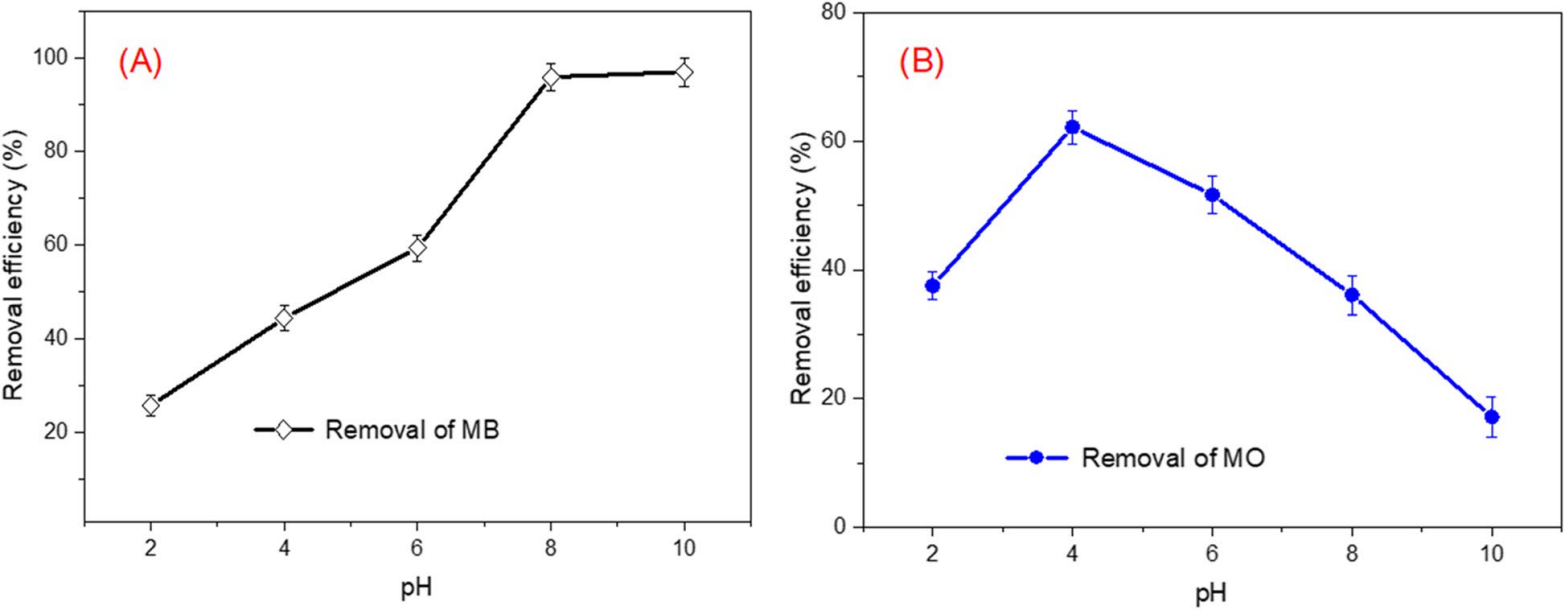
Effect of pH on the removal efficiency of MB (**A**) and MO (**B**) using zinc phosphate nanomaterial synthesized by *A. fumigatus* (initial dye concentration: 100 mg/L; solution volume: 100 ml; ZnP-dosage was 0.5 g (MB)-0.75 g (MO) temperature: 30 °C, agitation speed: 150 rpm; contact time = 120 min)

The initial solution pH value remarkably influences the whole adsorption process through the changes employed on the surface charge and the functional groups of the adsorbent particle’s active sites and the chemistry of the sorbate solution. The experimental results relating the influence of solution initial pH on the removal efficiency of MB and MO dyes by the biosynthetic ZnP-nanosheets was investigated at room temperature in the pH range 2–10 are shown in Fig. 8, while the other parameters were remained constant. From the results, it can deduce that the ZnP-nps sheets adsorption capacity for both cationic and anionic dyes is highly pH dependent. It is noticed that the removal efficiency of MB was slowly increased by increasing the initial pH of solution up to pH 8 and then become constant. The maximum removal of MO dye was determined at pH = 4.0 (62.1%). While the removal (%) of MO reduced in the pH range 5–10. The fluctuations in pH lead to a change of adsorbent surface which consequently affects the removal efficiency. The zero-point charge (pH_PZC_) of nanomaterial showed at pH = 6.9; when the pH was more than 6.9, the adsorbent surface was negatively charged. On contrary, when the pH was less than pH_PZC_, the surface of adsorbent appeared to be positively charged. At acidic pH, the surface of adsorbent is positively charged and the co-existed protons in the solution would increase, which endorsed the adsorption of anionic MO dye. In comparison at basic pH, the adsorbent surface is negatively charged and thereby generated an electrostatic repulsion with the OH^−^, adsorbent surface and anionic dye, which resulted in an increase in cationic MB dye adsorption. For further study, the pH 4.0 for MO and pH 8.0 for MB were selected as the optimum pH for cationic and anionic dyes adsorption, respectively. Similar results have also been mentioned by other researchers for cationic and anionic dyes removal using nanomaterials [67–70].

### Effect of initial MB and MO dyes concentration

**Fig. 9:**
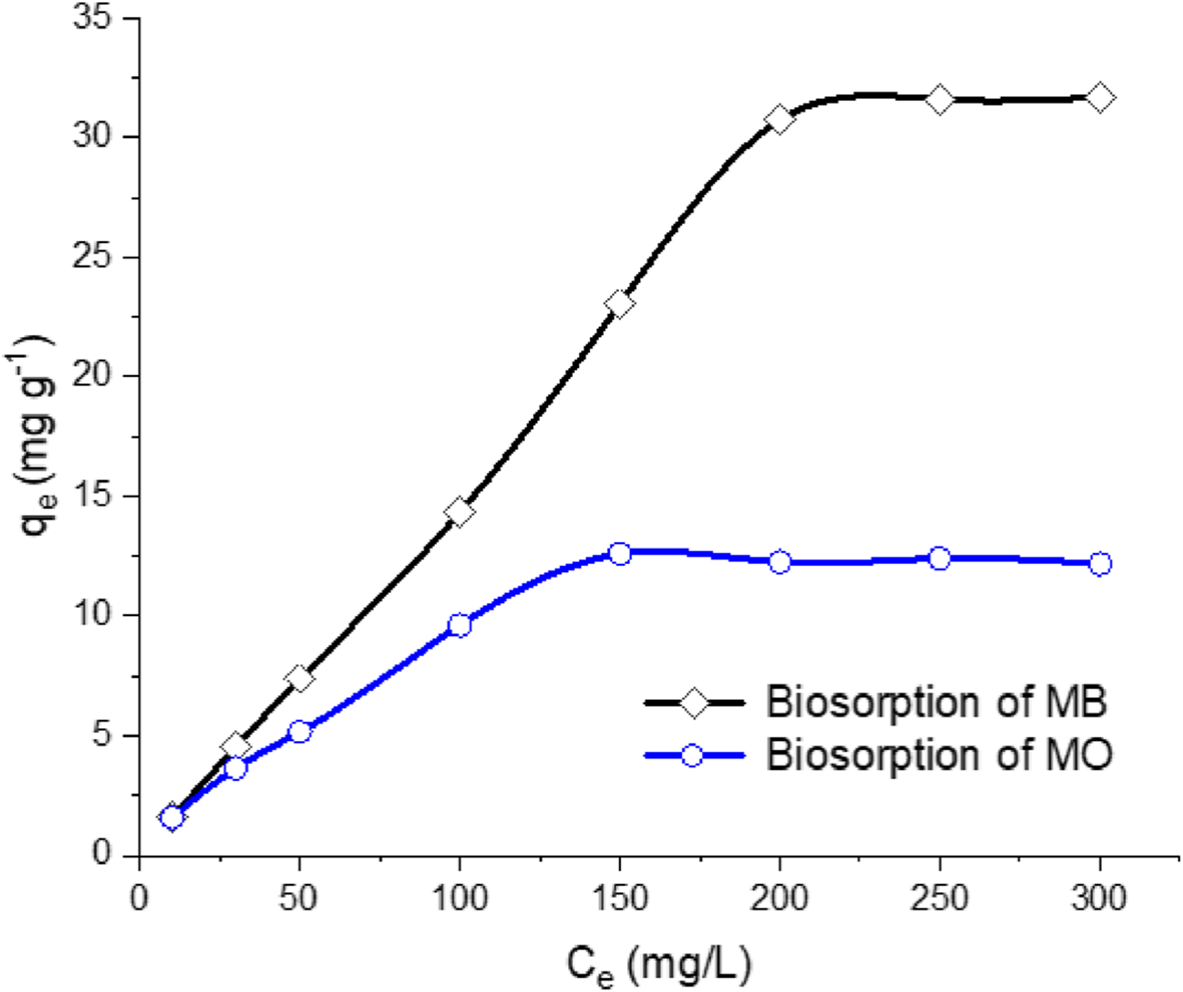
Effect of initial dye concentration on the MB and MO sorption using zinc phosphate nanosheets synthesized by *A. fumigatus* exo-metabolites (ZnP-dosage was 0.5 g (MB) and 0.75 g (MO); agitation speed: 150 rpm)

Sorption of various dyes by the biosynthetic zinc phosphate nanomaterial was investigated at various initial concentrations (10–300 mg/l) and the results were displayed in Fig. 9. Herein, the sorption process was enhanced by increasing the initial sorbent concentration, however, the removal rate reached equilibrium at the concentration of 200 mg/l and 150 mg/l for MB and MO, respectively. The probable reasons are that the initial biosorbent concentration provides a transfer driving force which contributes to outdo ions transfer struggle between the solid and aqueous phases. Meanwhile, the collision among the dye molecules and the sorbent was enhanced by increasing the initial sorbent concentration, which improves the sorption process [30, 71, 72].

### Sorption isotherms

Adsorption isotherms are considered as the most unique features for the design of adsorption process. The binding affinity and adsorption capacity were explored by studying the relation between the adsorbate concentration in solution and the quantity of sorbent required to remove pollutants. In the current study, the two common isotherm models of Langmuir and Freundlich were employed to the experimental data to characterize the adsorption behavior of MB and MO toward the biosynthetic zinc phosphate nanomaterial. The Langmuir’s isotherm assumes the monolayer adsorption of dye molecules onto the adsorbate whereas Freundlich’s isotherm describes multilayer adsorption of the dye molecules [73].

**Fig. 10:**
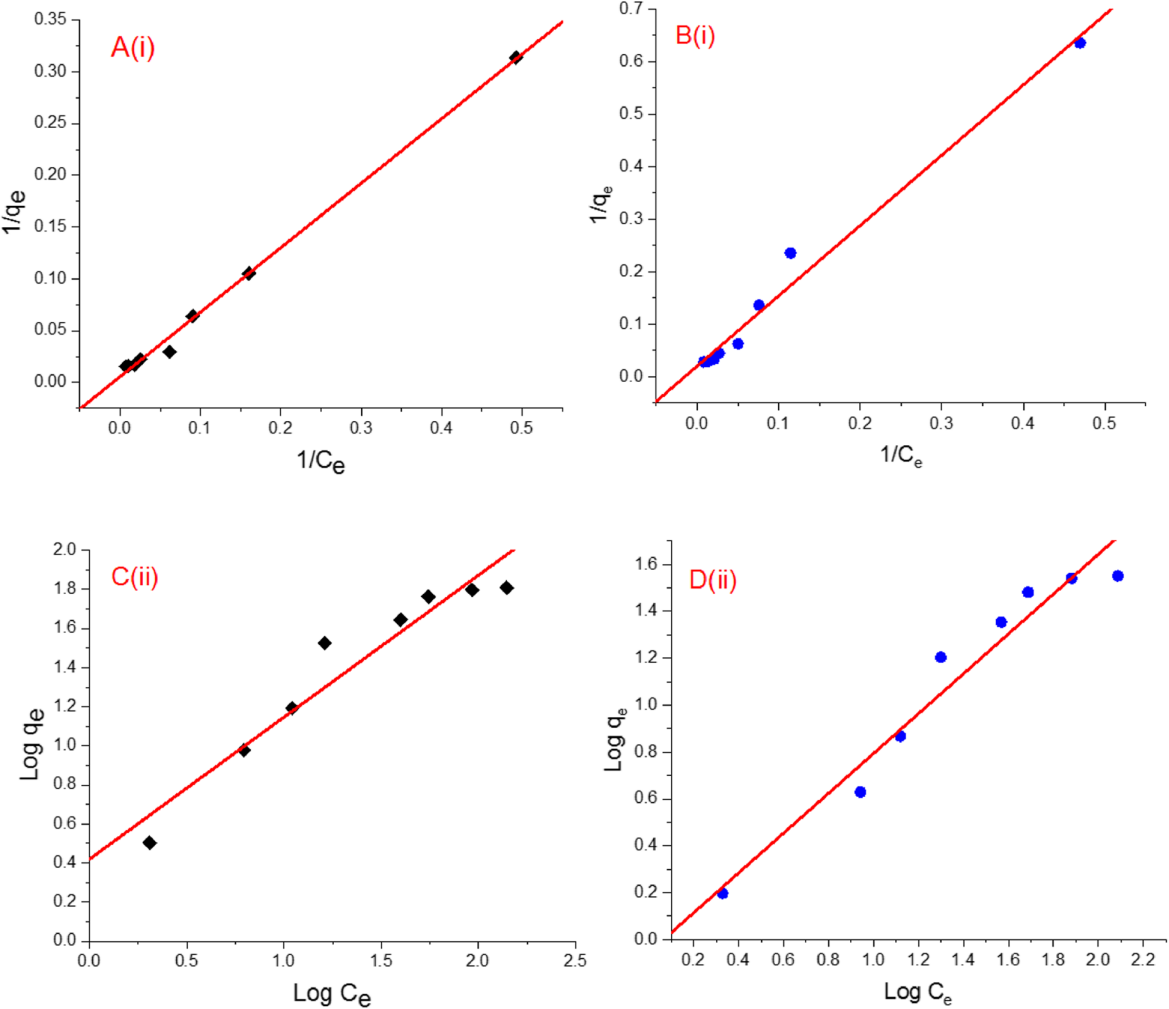
Adsorption isotherms of MB (**A**, **C**) and MO (**B**, **D**) sorbed by *A. fumigatus*-based zinc phosphate nanocomposite: (i) Langmuir and (ii) Freundlich

The linear plots of the Langmuir’s and Freundlich’s isotherms for MB and MO dyes adsorption by the zinc phosphate nanomaterial synthesized by *A. fumigatus* are shown in Fig. 10. High correlation coefficient (R^2^) was calculated for the two isotherms. The results suggest that both dyes show almost similar adsorption mechanism and Langmuir’s is more suitable than Freundlich’s isotherms for the adsorption of MB and MO dyes. Langmuir’s isotherm shows similar allocation of adsorption sites on the surface of adsorbate with each site has a similar enthalpy and activation energy [50]. The maximum values of adsorption capacity (q_max_) for MB and MO on nanosheets were 178.25 mg g^−1^ and 50.10 mg g^−1^, respectively (Table 1A). The Langmuir’s model showed lower *K*_*L*_ for MB and MO, indicating effective biosorption by nanomaterial. It can also be noticed that the high values of *K*_*F*_ and 1/n (n > 1), designating a great adsorptive capacity and easy adsorption of dye molecules.

**Table 1:** (**A**) Langmuir and Freundlich isotherms constants (**B**) Kinetic parameters for the biosorption of MB and MO onto the zinc phosphate nanosheets synthesized by *A. fumigatus*

A. Isotherm model	Equations	Parameters	Adsorbed dye	
			**MB**	**MO**
*Langmuir isotherm*	$\frac{1}{{q}_{e}}$ =$\frac{1}{{K}_{L}{q}_{max}} \frac{1}{{C}_{e}}+ \frac{1}{{q}_{max}}$	q_max_ (mg/g)	178.25	50.10
		*K*_*L*_ (L/mg)	0.01	0.04
		R^2^	0.99	0.99
*Freundlich isotherm*	q = K_*F*_ C^1/n^	K_F_ (mg/g)	2.632	0.877
		*n*	1.37	1.17
		R^2^	0.92	0.94
**B. Kinetic model**		q_e_ (exp.) (mg/g)	33.54	16.02
*Pseudo-first-order*	$\mathrm{ln}({q}_{e}-{q}_{t})= \mathrm{ln}{q}_{e}-{K}_{1}$ *t*	q_e_ (calc.) (mg/g)	7.54	3.55
		K_1_ (min^−1^)	0.116	0.125
		R^2^	0.904	0.617
*Pseudo-second-order*	$\frac{1}{{q}_{e}}$=$\frac{1}{{K}_{L } {q}_{max} {C}_{e}} \times \frac{1}{{q}_{max}}$	q_e_ (calc.) (mg/g)	33.61	16.13
		K_2_ (g/mg/min)	0.043	0.087
		R^2^	0.99	0.99

### Kinetics of MB and MO dyes adsorbed onto biosynthesized zinc phosphate nanosheets

**Fig. 11:**
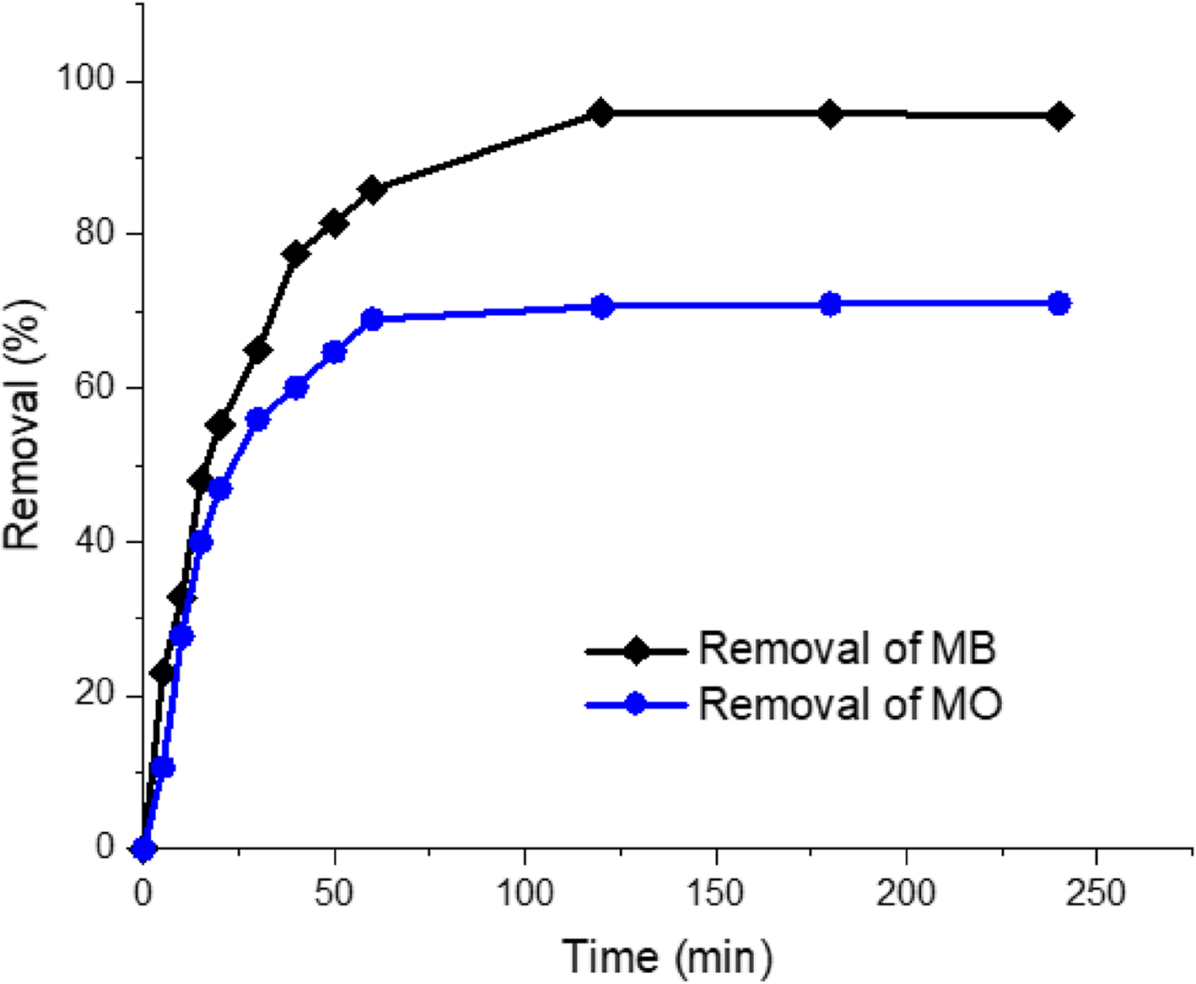
Effect of contact time on the sorption of MB and MO using zinc phosphate nanopowder synthesized by *A. fumigatus* (initial dye concentration: 100 mg/l; ZnP-dosage was 0.5 g (MB) and 0.75 g (MO); agitation speed: 150 rpm)

The variation in sorption capacity of cationic and anionic dyes with contact time was scrutinized using MB and MO at initial concentration of 100 mg/l. The contact time was varied in the range 5–240 min. As shown in Fig. 11, both MB and MO displayed similar trends during the variation in contact time. Most of the MB and MO are adsorbed using the fungal synthesized ZnP-nps in the first 50 min, whereas the maximum quantity of MB and MO adsorbed in a given time interval (q_e_) was detected at 120 min, beyond which the q_e_ attained almost plateau. Therefore, the contact time in subsequent experiments was kept at 120 min. Initially, the rapid sorption of dyes may be associated to the high affinity of nanomaterial toward MB and MO molecules and then become almost constant. In practical applications, the rapid sorption provides the ability to use smaller reactor volumes [71, 74]. Similar observations have been stated previously for various sorbent systems, in which firstly adsorption was sharply increased and subsequently reach equilibrium [28, 75, 76]. The time of equilibrium is highly reliant on the porosity and surface area of the adsorbent [50, 73].

**Fig. 12:**
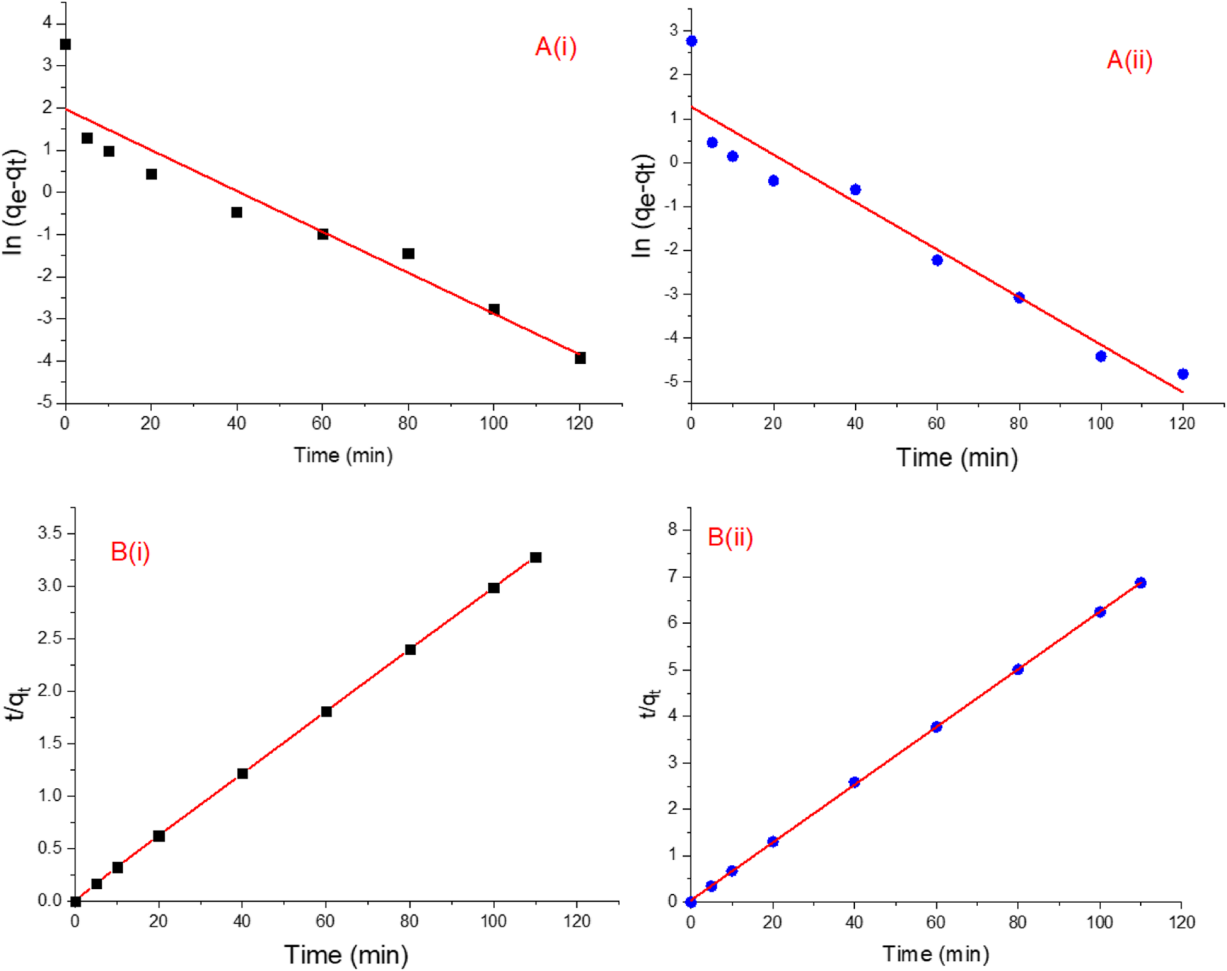
**A** Pseudo-first-order kinetics for the sorption of MB (i) and MO (ii) by zinc phosphate nanomaterial synthesized by *A. fumigatus* and **B** Pseudo-second-order kinetics for the sorption of MB (i) and MO (ii) by zinc phosphate nanomaterial synthesized by *A. fumigatus*

The sorption kinetics were estimated using the pseudo-first and second-order kinetic models and the results are presented in Fig. 12 and Table 1B. The sorption power calculated by pseudo-first order model displays poor fitting, regarding the linear regression coefficient (R^2^) and a significant alteration among calculated and experimental values have been detected. The sorption of individual cationic and anionic dyes were observed to effectively be expressed by a pseudo-2^nd^-order kinetic model as the extent of fitting (R^2^ > 0.999) were higher when compared with the pseudo-first-order dynamics model, assuming the adsorption of dyes molecules onto nanomaterial occurred via chemisorption and/or ion exchange mechanism [76, 77]. Moreover, the experimental q_e_ value is consistent with the calculated value of adsorption capacity. Our results agree with those previously described by [39, 73, 76] who mentioned that the adsorption of most dyes is best obeyed pseudo-2^nd^-order model.

### Reusability

**Fig. 13:**
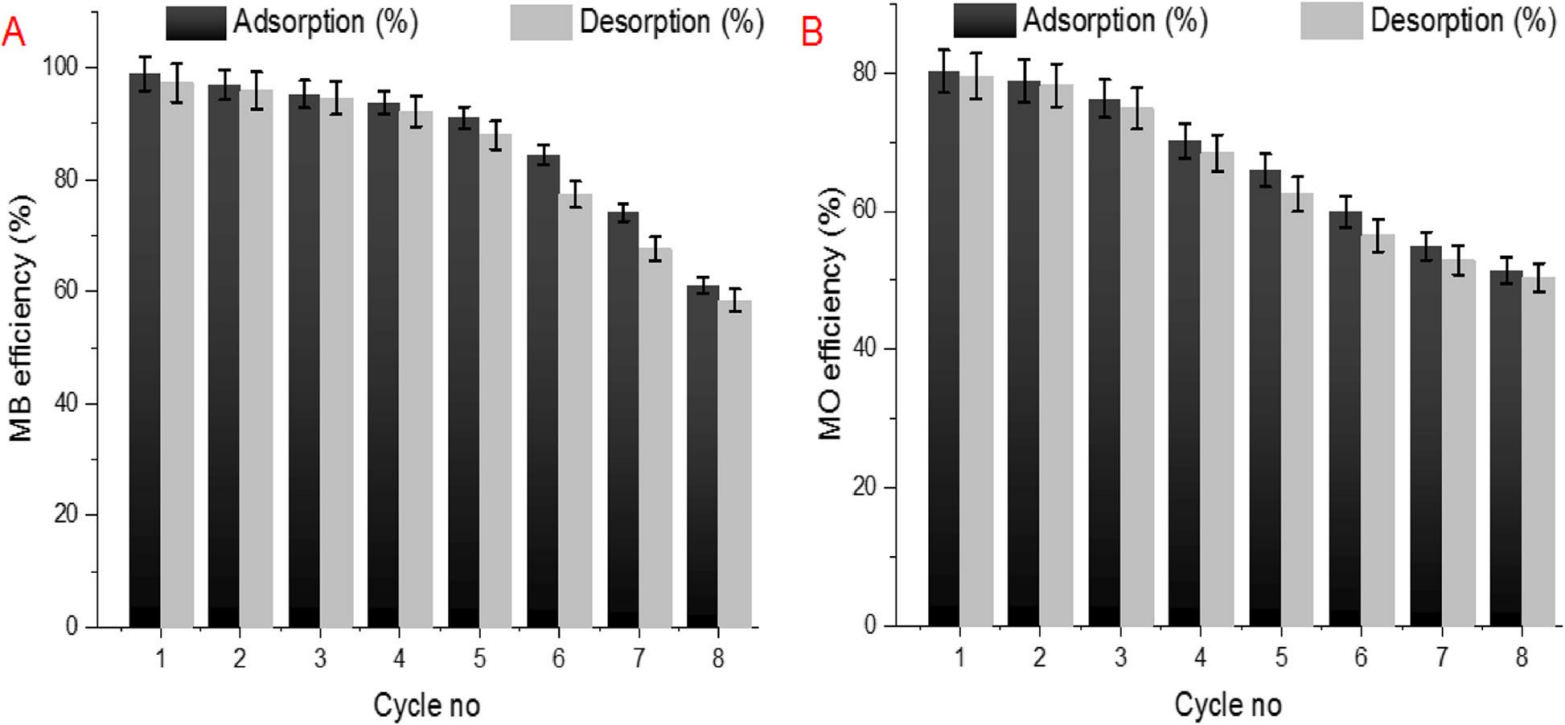
Reusability of the zinc phosphate nanosheets crystal in the removal of MB (**A**) and MO (**B**) during adsorption/desorption cycles

To investigate the effectiveness and sustainability of the *A. fumigatus*– based adsorbate, the reusability of nanomaterial was tested in 8 successive adsorption–desorption cycles using the stripping agent (0.1 M HCl). Initially, the adsorption capacity of ZnP nanosheets gradually decreased till 5^th^ cycle while retained 91.06 and 65.91% of MB and MO initial adsorption activity, respectively. This may be due to the activation of adsorbent pores by the desorbing agent. However, the destructive effect of stripping agent clearly appeared during the progress of adsorption/desorption cycles as shown in Fig. 13. Successful regeneration of various sorbent using acidic solution has been mentioned by other investigators [31, 78, 79].

### Comparison of zinc phosphate nanosheets synthesized by *A. fumigatus* with other sorbents

The adsorption capacity of the ZnP-nanomaterial synthesized using the fungal filtrate of *A. fumigatus* are related to those of other reported adsorbents in term of maximum adsorption capacity, isotherm, kinetic and reusability as shown in Table 2. The adsorption capacity of the prepared zinc phosphate nanomaterial toward MB and MO, in the present study, was superior over other previously engineered adsorbents. Hence, the ZnP-nanomaterial prepared using *A. fumigatus* exo-metabolites can be considered as an alternative ecofriendly adsorbent with sufficient removal activities toward textile anionic and cationic dyes.

**Table 2:** Comparison of adsorption capacity of the biosynthetic ZnP-nanosheets against various adsorbent

Dye	Adsorbent	q_m_ (mg/g)	Isotherm	Kinetic	Reuse	Reference
**A. Cationic dye**						
MB	Zinc phospahate nanosheets	178.25	L	PSO	+	**Current study**
	Fe_2_O_3_/SiO_2_ nanocomposite	116.09	L	PSO	+	Chen et al. 2016 [47]
	Co_3_O_4_/SiO_2_ nanocomposite	53.87	L	PSO	+	Abdel Ghafar et al. 2015 [51]
	Phosphoric acid based geopolymers	4.2	L	PSO	+	Khan et al. 2015 [50]
**B. Anionic dye**						
	Zinc phospahate nanosheets	50.1	L	PSO	+	**Current study**
MO	TiO_2_ nanocomposite	42.85	L	PSO	+	Ahmad et al. 2017 [52]
	Ferric oxide–biochar nano-composites	20.53	L	PSO	+	Chaukura et al. 2017 [53]
	iron oxide/carbon nanocomposites	32.63	L	PSO	+	Istratie et al. 2019 [54]

^*^L Langmuir isotherm, *PSO* pseudo-second-order kinetic,—= not reused

## Conclusions

*A. fumigatus* can synthesis a non-toxic, cost-effective, and eco-friendly zinc phosphate nanosheets crystal as confirmed by XRD, EDX, SEM, TEM, and FTIR analyses. The obtained results marked the potential of the biosynthetic ZnP-nanomaterial as in vitro antibacterial agent against Gram-positive and Gram–negative bacterial pathogens. Further studies are required for testing of their potential applications as alternative to conventional antibiotics and in drug development. The antiradical scavenging ability of ZnP-based nanomaterial was found to be in a concentration dependent manner. The sorption capacity of the biosynthetic nanomaterial was examined in the presence of anionic and cationic textile dyes and exhibited superior adsorptive activity and can be recycled several times. Therefore, *A. fumigatus*-mediated zinc phosphate nanosheets synthesis could be considered as a sustainable, and new efficient adsorbent for removing the toxic textile dyes from wastewater with lower environmental impact. However, further research is required for the movement of the lab scale study to the real-world, including the application of nanosheets in industrial scale for real water dye removal/wastewater treatment process and for other environmental treatments. The recycling and regeneration are ZnP-based nanomaterial is of great prominence for its sustainability, however, this part desires additional research due to the effectiveness of certain regenerated nanosheets are not well restored and may produce secondary contamination. To the best of our knowledge, there is not any report regarding the green synthesis of zinc nanosheets-based phosphates by filamentous fungus or its significant capability in the dye decolorization.

### Supplementary Information

**Additional file 1.**

## Data Availability

The data generated or analyzed in this investigation are included in this article file and its supplementary information.

## References

[1] Zhu C, Yang G, Li H, Du D, Lin Y (2015). Electrochemical sensors and biosensors based on nanomaterials and nanostructures. Anal Chem.

[2] Kociova S, Dolezelikova K, Horky P, Skalickova S, Baholet D, Bozdechova L (2020). Zinc phosphate-based nanoparticles as alternatives to zinc oxide in diet of weaned piglets. J Anim Sci Biotechnol.

[3] Mohd Yusof H, Rahman A, Mohamad R, Zaidan UH, Samsudin AA (2020). Biosynthesis of zinc oxide nanoparticles by cell-biomass and supernatant of *Lactobacillus plantarum* TA4 and its antibacterial and biocompatibility properties. Sci Rep.

[4] El-Shora HM, Khateb AM, Darwish DB, El-Sharkawy RM (2022). Thiolation of myco-synthesized Fe_3_O_4_-NPs: a novel promising tool for *Penicillium Expansium* laccase immobilization to decolorize textile dyes and as an application for anticancer agent. J Fungi.

[5] El-Sharkawy RM, Swelim MA, Hamdy GB (2022). *Aspergillus tamarii* mediated green synthesis of magnetic chitosan beads for sustainable remediation of wastewater contaminants. Sci Rep.

[6] Al-Radadi NS, Hussain T, Faisal S, Shah SAR (2022). Novel biosynthesis, characterization and bio-catalytic potential of green algae (*Spirogyra hyalina*) mediated silver nanomaterials. Saudi J Biol Sci.

[7] Faisal S, Abdullah, Jan H, Shah SA, Shah S, Rizwan M (2021). Bio-catalytic activity of novel *Mentha arvensis* intervened biocompatible magnesium oxide nanomaterials. Catalysts.

[8] Faisal S, Al-Radadi NS, Jan H, Abdullah, Shah SA, Shah S (2021). *Curcuma longa* mediated synthesis of copper oxide, nickel oxide and Cu-Ni bimetallic hybrid nanoparticles: characterization and evaluation for antimicrobial, anti-parasitic and cytotoxic potentials. Coatings.

[9] Faisal S, Jan H, Shah SA, Shah S, Khan A, Akbar MT (2021). Green synthesis of zinc oxide (ZnO) nanoparticles using aqueous fruit extracts of *Myristica fragrans*: their characterizations and biological and environmental applications. ACS Omega.

[10] Faisal S, Khan S, Abdullah, Zafar S, Rizwan M, Ali M (2022). *Fagonia Cretica*-mediated synthesis of manganese oxide (MnO_2_) nanomaterials their characterization and evaluation of their bio-catalytic and enzyme inhibition potential for maintaining flavor and texture in apples. Catalysts.

[11] Shah S, Shah SA, Faisal S, Khan A, Ullah R, Ali N (2022). Engineering novel gold nanoparticles using *Sageretia thea* leaf extract and evaluation of their biological activities. J Nanostructure Chem.

[12] Abdullah R, Au, Faisal S, Almostafa MM, Younis NS, Yahya G (2023). Multifunctional Spirogyra-hyalina-mediated Barium Oxide nanoparticles (BaONPs): synthesis and applications. Molecules.

[13] Faisal S, Rizwan M, Ullah R, Alotaibi A, Khattak A, Bibi N et al. Paraclostridium benzoelyticum bacterium-mediated zinc oxide nanoparticles and their in vivo multiple biological applications. Oxid Med Cell Longev. 2022;2022:15. Article ID 5994033. 10.1155/2022/5994033 PMC9098347 35571251

[14] Faisal S, Ullah R, Alotaibi A, Zafar S, Rizwan M, Tariq MH (2023). Biofabrication of silver nanoparticles employing biomolecules of paraclostridium benzoelyticum strain: its characterization and their in-vitro antibacterial, anti-aging, anti-cancer and other biomedical applications. Microsc Res Tech.

[15] Hussain T, Faisal S, Rizwan M, Zaman N, Iqbal M, Iqbal A (2022). Green synthesis and characterization of copper and nickel hybrid nanomaterials: investigation of their biological and photocatalytic potential for the removal of organic crystal violet dye. J Saudi Chem Soc.

[16] Ullah R, Shah S, Muhammad Z, Shah SA, Faisal S, Khattak U (2021). In vitro and in vivo applications of Euphorbia wallichii shoot extract-mediated gold nanospheres. Green Process Synth.

[17] Zafar S, Faisal S, Jan H, Ullah R, Rizwan M, Abdullah (2022). Development of Iron nanoparticles (FeNPs) using biomass of *enterobacter*: its characterization, antimicrobial, Anti-alzheimer’s, and enzyme inhibition potential. Micromachines.

[18] El-Sharkawy R, Alshora HM (2020). Biocontrol of wilt-inducing *Fusarium oxysporum* by aqueous leaf extract from Egyptian *Ammi majus* and *Ammi visnaga*. Egypt J Bot.

[19] EL-Shora HM, El-Sharkawy RM. Evaluation of putative inducers and inhibitors toward tyrosinase from two Trichoderma species. Jordan J Biolo Sci. 2020;13(1).

[20] Horky P, Skalickova S, Urbankova L, Baholet D, Kociova S, Bytesnikova Z (2019). Zinc phosphate-based nanoparticles as a novel antibacterial agent: *in vivo* study on rats after dietary exposure. J Anim Sci Biotechnol.

[21] Ansar S, Tabassum H, Aladwan NS, Naiman Ali M, Almaarik B, AlMahrouqi S (2020). Eco friendly silver nanoparticles synthesis by *Brassica oleracea* and its antibacterial, anticancer and antioxidant properties. Sci Rep.

[22] Bhakya S, Muthukrishnan S, Sukumaran M, Muthukumar M (2016). Biogenic synthesis of silver nanoparticles and their antioxidant and antibacterial activity. Appl Nanosci.

[23] Nicola R, Costişor O, Muntean S-G, Nistor M-A, Putz A-M, Ianăşi C (2020). Mesoporous magnetic nanocomposites: a promising adsorbent for the removal of dyes from aqueous solutions. J Porous Mater.

[24] Yazdani MR, Virolainen E, Conley K, Vahala R (2018). Chitosan–Zinc (II) complexes as a bio-sorbent for the adsorptive abatement of phosphate: mechanism of complexation and assessment of adsorption performance. Polymers.

[25] Chaukura N, Murimba EC, Gwenzi W (2017). Synthesis, characterisation and methyl orange adsorption capacity of ferric oxide–biochar nano-composites derived from pulp and paper sludge. Appl Water Sci.

[26] Kaviyarasu K, Geetha N, Kanimozhi K, Magdalane CM, Sivaranjani S, Ayeshamariam A (2017). In vitro cytotoxicity effect and antibacterial performance of human lung epithelial cells A549 activity of zinc oxide doped TiO_2_ nanocrystals: investigation of bio-medical application by chemical method. Mater Sci Eng: C.

[27] Isa N, Lockman Z (2019). Methylene blue dye removal on silver nanoparticles reduced by *Kyllinga brevifolia*. Environ Sci Pollut Res.

[28] Nguyen TH, Watari T, Hatamoto M, Sutani D, Setiadi T, Yamaguchi T (2020). Evaluation of a combined anaerobic baffled reactor–downflow hanging sponge biosystem for treatment of synthetic dyeing wastewater. Environ Technol Innov.

[29] Srivastava A, Dangi LK, Kumar S, Rani R (2022). Microbial decolorization of reactive black 5 dye by Bacillus albus DD1 isolated from textile water effluent: kinetic, thermodynamics and decolorization mechanism. Heliyon..

[30] Fomina M, Gadd GM (2014). Biosorption: current perspectives on concept, definition and application. Bioresour Technol.

[31] Abate GY, Alene AN, Habte AT, Addis YA (2021). Adsorptive removal of basic green dye from aqueous solution using humic acid modified magnetite nanoparticles: kinetics, equilibrium and thermodynamic studies. J Polym Environ.

[32] Georgin J, Franco DS, Netto MS, Allasia D, Oliveira ML, Dotto GL (2020). Treatment of water containing methylene by biosorption using Brazilian berry seeds (*Eugenia uniflora*). Environ Sci Pollut Res.

[33] Torres E, Biosorption. A review of the latest advances. Proc. 2020;8(12):1584.

[34] Giese EC, Silva DD, Costa AF, Almeida SG, Dussán KJ (2020). Immobilized microbial nanoparticles for biosorption. Crit Rev Biotechnol.

[35] Sintakindi A, Ankamwar B (2021). Fungal biosorption as an alternative for the treatment of dyes in waste waters: a review. Environ Technol Rev.

[36] Ahmad A, Khan N, Giri BS, Chowdhary P, Chaturvedi P (2020). Removal of methylene blue dye using rice husk, cow dung and sludge biochar: characterization, application, and kinetic studies. Bioresour Technol.

[37] Bayomie OS, Kandeel H, Shoeib T, Yang H, Youssef N, El-Sayed MM (2020). Novel approach for effective removal of methylene blue dye from water using fava bean peel waste. Sci Rep.

[38] Singh S, Kumar A, Gupta H (2020). Activated banana peel carbon: a potential adsorbent for rhodamine B decontamination from aqueous system. App Water Sci.

[39] Ali I, Peng C, Khan ZM, Sultan M, Naz I (2018). Green synthesis of phytogenic magnetic nanoparticles and their applications in the adsorptive removal of crystal violet from aqueous solution. Arab J Sci Eng.

[40] Benvenuti J, Giraldi Fisch A, Zimnoch Dos Santos JH, Gutterres M (2020). Hybrid sol–gel silica adsorbent material based on grape stalk applied to cationic dye removal. Environ Progr Sustain Energy.

[41] Raper KB, Fennell DI. The genus *aspergillus*. The Genus Aspergillus. 1965.

[42] El-Shora HM, El-Sharkawy RM, Khateb AM, Darwish DB (2021). Production and immobilization of β-glucanase from *Aspergillus Niger* with its applications in bioethanol production and biocontrol of phytopathogenic fungi. Sci Rep.

[43] El-Shora HM, El-Sharkawy RM (2020). Tyrosinase from *Penicillium Chrysogenum*: characterization and application in phenol removal from aqueous solution. J Gen Appl Microbiol.

[44] Zhou X, Bai H, Ma H, Li H, Yuan W, Du H (2015). Synthesis of zinc phosphate and zinc ammonium phosphate nanostructures with different morphologies through pH control. Mater Charact.

[45] Qais FA, Shafiq A, Khan HM, Husain FM, Khan RA, Alenazi B (2019). Antibacterial effect of silver nanoparticles synthesized using Murraya koenigii (L.) against multidrug-resistant pathogens. Bioinorg Chem Appl.

[46] Singh PK, Bhardwaj K, Dubey P, Prabhune A (2015). UV-assisted size sampling and antibacterial screening of *Lantana camara* leaf extract synthesized silver nanoparticles. RSC adv.

[47] Yamaguchi Y, Kahle AB, Tsu H, Kawakami T, Pniel M (1998). Overview of advanced spaceborne thermal emission and reflection radiometer (ASTER). IEEE Trans Geosci Remote Sens.

[48] Awah FM (2010). Antioxidant activity, nitric oxide scavenging activity and phenolic contents of *Ocimum gratissimum* leaf extract. J Medic Plant Res.

[49] Keshari AK, Srivastava A, Verma AK, Srivastava R (2016). Free radicals scavenging and protein protective property of *Ocimum sanctum* (L). Br J Pharm Res.

[50] Khan MI, Min TK, Azizli K, Sufian S, Ullah H, Man Z (2015). Effective removal of methylene blue from water using phosphoric acid based geopolymers: synthesis, characterizations and adsorption studies. RSC adv.

[51] Sadeghi-Aghbash M, Rahimnejad M, Pourali SM (2020). Bio-mediated synthesis and characterization of zinc phosphate nanoparticles using *Enterobacter aerogenes* cells for antibacterial and anti-corrosion applications. Curr Pharm Biotechnol.

[52] Tian W, Yang L-M, Xu Y-Z, Weng S-F, Wu J-G (2000). Sugar interaction with metal ions. FT-IR study on the structure of crystalline galactaric acid and its K^+^, NH^4+^, Ca^2+^, Ba^2+^, and La^3+^ complexes. Carbohydr Res.

[53] He W, Yan S, Wang Y, Zhang X, Zhou W, Tian X (2009). Biomimetic synthesis of mesoporous zinc phosphate nanoparticles. J Alloys Compd.

[54] Zhan Q, Qian C (2016). Microbial-induced synthesis of nanoparticles of zinc phosphate and basic zinc carbonate based on the degradation of glyphosate. Dig J Nanomater Biostruct.

[55] Bach S, Celinski VR, Dietzsch M, Panthöfer M, Bienert R, Emmerling F (2015). Thermally highly stable amorphous zinc phosphate intermediates during the formation of zinc phosphate hydrate. J Am Chem Soc.

[56] Yue D, Lu W, Li C, Zhang X, Liu C, Wang Z (2014). Controllable synthesis of Ln3+(ln = Tb, Eu) doped zinc phosphate nano-/micro-structured materials: phase, morphology and luminescence properties. Nanoscale.

[57] Roy A, Bulut O, Some S, Mandal AK, Yilmaz MD (2019). Green synthesis of silver nanoparticles: biomolecule-nanoparticle organizations targeting antimicrobial activity. RSC adv.

[58] Almoudi MM, Hussein AS, Hassan MIA, Zain NM (2018). A systematic review on antibacterial activity of zinc against Streptococcus mutans. Saudi Dent J.

[59] Zuo K, Yin Y, Yao L, Wang K, Yan Y, Ma Z (2020). Antibacterial activities and cytocompatibility of zinc-contained strontium phosphate coating on titanium. Mater Res Express.

[60] Dakal TC, Kumar A, Majumdar RS, Yadav V (2016). Mechanistic basis of antimicrobial actions of silver nanoparticles. Front Microbiol.

[61] Shankar S, Rhim J-W (2017). Facile approach for large-scale production of metal and metal oxide nanoparticles and preparation of antibacterial cotton pads. Carbohydr Polym.

[62] Fatima N, Qazi UY, Mansha A, Bhatti IA, Javaid R, Abbas Q (2021). Recent developments for antimicrobial applications of graphene-based polymeric composites: a review. J Ind Eng Chem.

[63] Murali M, Mahendra C, Rajashekar N, Sudarshana M, Raveesha K, Amruthesh K (2017). Antibacterial and antioxidant properties of biosynthesized zinc oxide nanoparticles from *Ceropegia candelabrum* L.–an endemic species. Spectrochim Acta Part A Mol Biomol Spectrosc.

[64] de Oliveira KJF, Donangelo CM, de Oliveira AV, da Silveira CLP, Koury JC (2009). Effect of zinc supplementation on the antioxidant, copper, and iron status of physically active adolescents. Cell Biochem Function: Cell Biochem its Modulation Act Agents or Disease.

[65] Ibrahim S, Ahmad Z, Manzoor MZ, Mujahid M, Faheem Z, Adnan A (2021). Optimization for biogenic microbial synthesis of silver nanoparticles through response surface methodology, characterization, their antimicrobial, antioxidant, and catalytic potential. Sci Rep.

[66] Sun W, Sun W, Wang Y (2019). Biosorption of direct fast scarlet 4BS from aqueous solution using the green-tide-causing marine algae Enteromorpha prolifera. Spectrochim Acta Part A Mol Biomol Spectrosc.

[67] Shabaan OA, Jahin HS, Mohamed GG (2020). Removal of anionic and cationic dyes from wastewater by adsorption using multiwall carbon nanotubes. Arab J Chem.

[68] Reddy DHK, Lee S-M (2013). Application of magnetic chitosan composites for the removal of toxic metal and dyes from aqueous solutions. Adv Colloid Interface Sci.

[69] Zafar MN, Dar Q, Nawaz F, Zafar MN, Iqbal M, Nazar MF (2019). Effective adsorptive removal of azo dyes over spherical ZnO nanoparticles. J Mater Res Technol.

[70] de Araújo TP, Tavares FO, Vareschini DT, Barros MAS (2021). Biosorption mechanisms of cationic and anionic dyes in a low-cost residue from brewer’s spent grain. Environmen Technol.

[71] Aksu Z (2001). Equilibrium and kinetic modelling of cadmium (II) biosorption by *C. Vulgaris* in a batch system: effect of temperature. Sep Purif Technol.

[72] Barka N, Abdennouri M, El Makhfouk M, Qourzal S (2013). Biosorption characteristics of cadmium and lead onto eco-friendly dried cactus (*Opuntia ficus indica*) cladodes. J Environ Chem Eng.

[73] Abd-Elhamid A, Emran M, El-Sadek M, El-Shanshory AA, Soliman H, Akl M (2020). Enhanced removal of cationic dye by eco-friendly activated biochar derived from rice straw. Appl Water Sci.

[74] Liu L, Zhang B, Zhang Y, He Y, Huang L, Tan S (2015). Simultaneous removal of cationic and anionic dyes from environmental water using montmorillonite-pillared graphene oxide. J Chem Eng Data.

[75] Hassan SE-D, Fouda A, Radwan AA, Salem SS, Barghoth MG, Awad MA (2019). Endophytic actinomycetes *Streptomyces* spp mediated biosynthesis of copper oxide nanoparticles as a promising tool for biotechnological applications. JBIC J Biological Inorg Chem.

[76] Pandey G, Singh S, Hitkari G (2018). Synthesis and characterization of polyvinyl pyrrolidone (PVP)-coated Fe_3_O_4_ nanoparticles by chemical co-precipitation method and removal of Congo red dye by adsorption process. Int Nano Lett.

[77] Cheera P, Karlapudi S, Sellola G, Ponneri V (2016). A facile green synthesis of spherical Fe_3_O_4_ magnetic nanoparticles and their effect on degradation of methylene blue in aqueous solution. J Mol Liq.

[78] Kupai J, Razali M, Buyuktiryaki S, Kecili R, Szekely G (2017). Long-term stability and reusability of molecularly imprinted polymers. Polym Chem.

[79] Xia X, Zhou Z, Wu S, Wang D, Zheng S, Wang G. Adsorption removal of multiple dyes using biogenic selenium nanoparticles from an *Escherichia coli* strain overexpressed selenite reductase CsrF. Nanomater. 2018;8(4):234. 10.3390/nano8040234 PMC5923564 29649129

