# Supplementary material for: Unveiling antibacterial and antioxidant activities of zinc phosphate-based nanosheets synthesized by Aspergillus fumigatus and its application in sustainable decolorization of textile wastewater
Source: BMC Microbiol. 2023 Nov 18;23:358. doi: 10.1186/s12866-023-03054-x (PMC10657121; doi:10.1186/s12866-023-03054-x)

**Unveiling antibacterial and antioxidant activities of zinc phosphate-based nanosheets synthesized by *Aspergillus fumigatus* and its application in sustainable decolorization of textile wastewater**

**Reyad M. El-Sharkawy^1^*, Mohamed H.H. Abbas^2^**

^1^Botany and Microbiology Department, Faculty of Science, Benha University, Benha 13511, Egypt

^2^Soils and Water Department, Faculty of Science, Benha University, Benha, Egypt

* Correspondence: [r.m.elsharkawy@fsc.bu.edu.eg](mailto:r.m.elsharkawy@fsc.bu.edu.eg), <https://orcid.org/0000-0003-1319-9066>

**Author information**

**Affiliations**

**Botany and Microbiology Department, Faculty of Science, Benha University, Benha 13511, Egypt;**

Reyad M. El-Sharkawy, [r.m.elsharkawy@fsc.bu.edu.eg](mailto:r.m.elsharkawy@fsc.bu.edu.eg)

**Soils and Water Department, Faculty of Science, Benha University, Benha, Egypt**

**Mohamed H.H. Abbas,** [mohamed.abbas@fagr.bu.edu.eg](mailto:mohamed.abbas@fagr.bu.edu.eg)

**Corresponding authors**

Correspondence to Reyad M. El-Sharkawy, email**:** [r.m.elsharkawy@fsc.bu.edu.eg](mailto:r.m.elsharkawy@fsc.bu.edu.eg), <https://orcid.org/0000-0003-1319-9066>

**Fig. S1** Uncropped gel image of PCR product in Figure 1A.


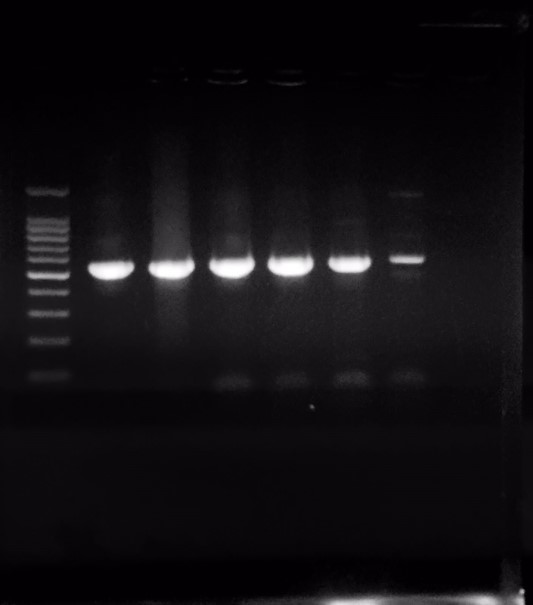

Supplement: Supplementary file 1 — Additional file 1. [file 12866_2023_3054_MOESM1_ESM.docx]
